# Ocular adverse events associated with immune checkpoint inhibitors, a scoping review

**DOI:** 10.1186/s12348-022-00321-2

**Published:** 2023-02-22

**Authors:** A. Martens, P. P. Schauwvlieghe, A. Madoe, I. Casteels, S. Aspeslagh

**Affiliations:** 1 Department of Ophthalmology, University Hospitals Leuven, Louvain, Belgium; 2 Department of Medical Oncology, University Hospital Brussels, Brussels, Belgium

**Keywords:** Immune checkpoint inhibitors, Immune related adverse event, Paraneoplastic, Eye

## Abstract

**Introduction:**

Immune checkpoint inhibitors (ICIs) have become an important part of the treatment of multiple cancers, especially for advanced melanoma and non-small cell lung cancer. Some tumors are capable of escaping immunosurveillance by stimulating checkpoints on T-cells. ICIs prevent activation of these checkpoints and thereby stimulate the immune system and indirectly the anti-tumor response. However, the use of ICIs is associated with various adverse events. Ocular side effects are rare but may have a major impact on the quality of life of the patient.

**Methods:**

A comprehensive literature search of the medical databases Web of Science, Embase and PubMed was performed. Articles that provided a comprehensive description of a case report containing 1) cancer patient(s) treated with (a combination of) immune checkpoint inhibitors, and 2) assessed occurrence of ocular adverse events, were included. A total of 290 case reports were included.

**Results:**

Melanoma (*n* = 179; 61.7%) and lung cancer (*n* = 56; 19.3%) were the most frequent reported malignancies. The primary used ICIs were nivolumab (*n* = 123; 42.5%) and ipilimumab (*n* = 116; 40.0%). Uveitis was most the common adverse event (*n* = 134; 46.2%) and mainly related to melanoma. Neuro-ophthalmic disorders, including myasthenia gravis and cranial nerve disorders, were the second most common adverse events (*n* = 71; 24.5%), mainly related to lung cancer. Adverse events affecting the orbit and the cornea were reported in 33 (11.4%) and 30 cases (10.3%) respectively. Adverse events concerning the retina were reported in 26 cases (9.0%).

**Conclusion:**

The aim of this paper is to provide an overview of all reported ocular adverse events related to the use of ICIs. The insights retrieved from this review might contribute to a better understanding of the underlying mechanisms of these ocular adverse events. Particularly, the difference between actual immune-related adverse events and paraneoplastic syndromes might be relevant. These findings might be of great value in establishing guidelines on how to manage ocular adverse events related to ICIs.

**Supplementary Information:**

The online version contains supplementary material available at 10.1186/s12348-022-00321-2.

## Introduction

**Fig. 1:**
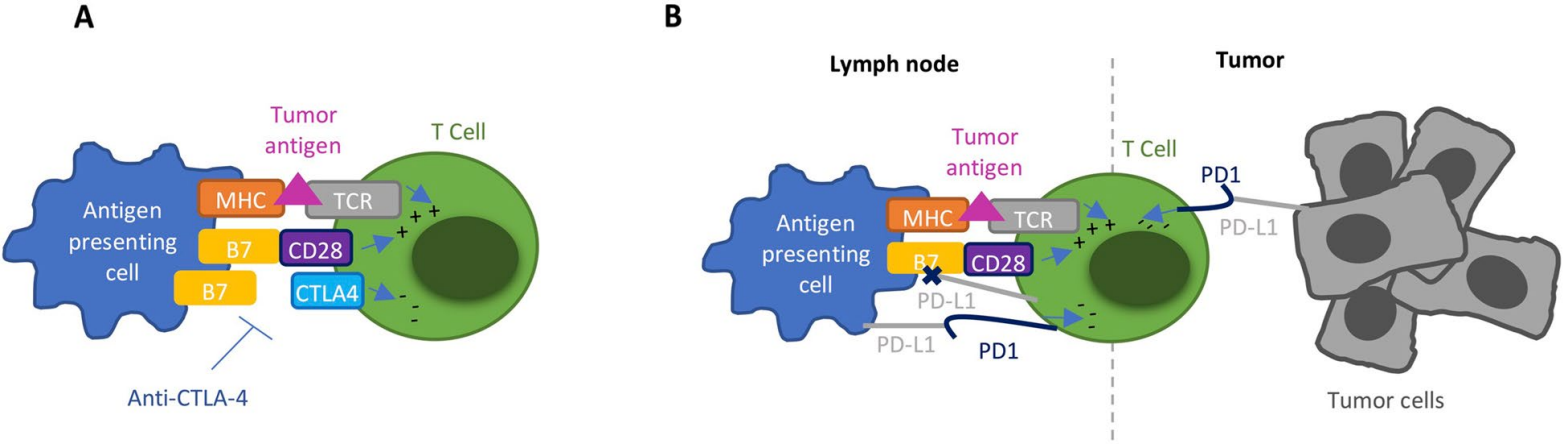
Mechanism of action. **A** Tumor antigen presentation by the major histocompatibility complex (MHC) receptor and B7/CD28 costimulatory signal are both necessary for CD8 T-cell activation. CTLA-4 downregulates T-cell immune function. Anti-CTLA-4 antibodies inhibit this downregulation and stimulate the immune system. **B** Interaction between PD1 and PD-L1 takes place in the lymph node and in the tumor microenvironment. PD1 downregulates T-cell function as well. Trough PD-L1 binding PD1, tumors can escape immunosurveillance. Anti-PD-1 or -PD-L1 antibodies prohibit this and will restore the anti-tumor response

Immune checkpoint inhibitors (ICIs) have led to a revolution in the treatment of multiple cancers, especially for advanced melanoma and non-small cell lung cancer (NSCLC) [1]. Some tumors are capable of escaping immunosurveillance by stimulating checkpoints on T-cells. By activating these checkpoints, tumors can escape the cellular immune reaction and can survive and spread. ICIs prevent activation of these checkpoints and thereby stimulate our immune system to attack tumor cells [2, 3]. Ipilimumab and tremelimumab are both human immunoglobulin G (IgG) antibodies against cytotoxic T-lymphocyte-associated antigen 4 (CTLA-4). Nivolumab, pembrolizumab, cemiplimab and sintilimab are IgG4 monoclonal antibodies against programmed cell death (PD-1) protein. CTLA-4 and PD-1 are both found on the surface of T-cells. Atezolizumab, avelumab and durvalumab are all IgG1 monoclonal antibodies against protein programmed death-ligand 1 (PD-L1). PD-L1 is produced by tumor cells to inhibit T-cells trough binding on the PD-1 receptor (Fig. 1). These ICIs are both used in monotherapy, as in combination [1, 4, 5]. The use of ICIs is expanding because of the remarkable results in terms of anti-tumor efficacy. However, multiple side-effects related to the use of ICIs have been reported, often referred to as immune-related adverse events (irAEs). Overall, ocular side effects are not common. It is thought that 1% of the patients treated with ICIs develop ocular side effects [3, 5]. However, recent studies showed that incidences may vary between 2.8 and 4.3% [6–8]. In this paper, the aim is to identify and analyze all ocular adverse events related to the use of ICIs. This overview might raise awareness among oncologists and ophthalmologists regarding the possible ocular side effects of ICIs and how to manage them. In addition, this review might contribute to a better understanding of the mechanisms of adverse events.

## Methods

**Fig. 2:**
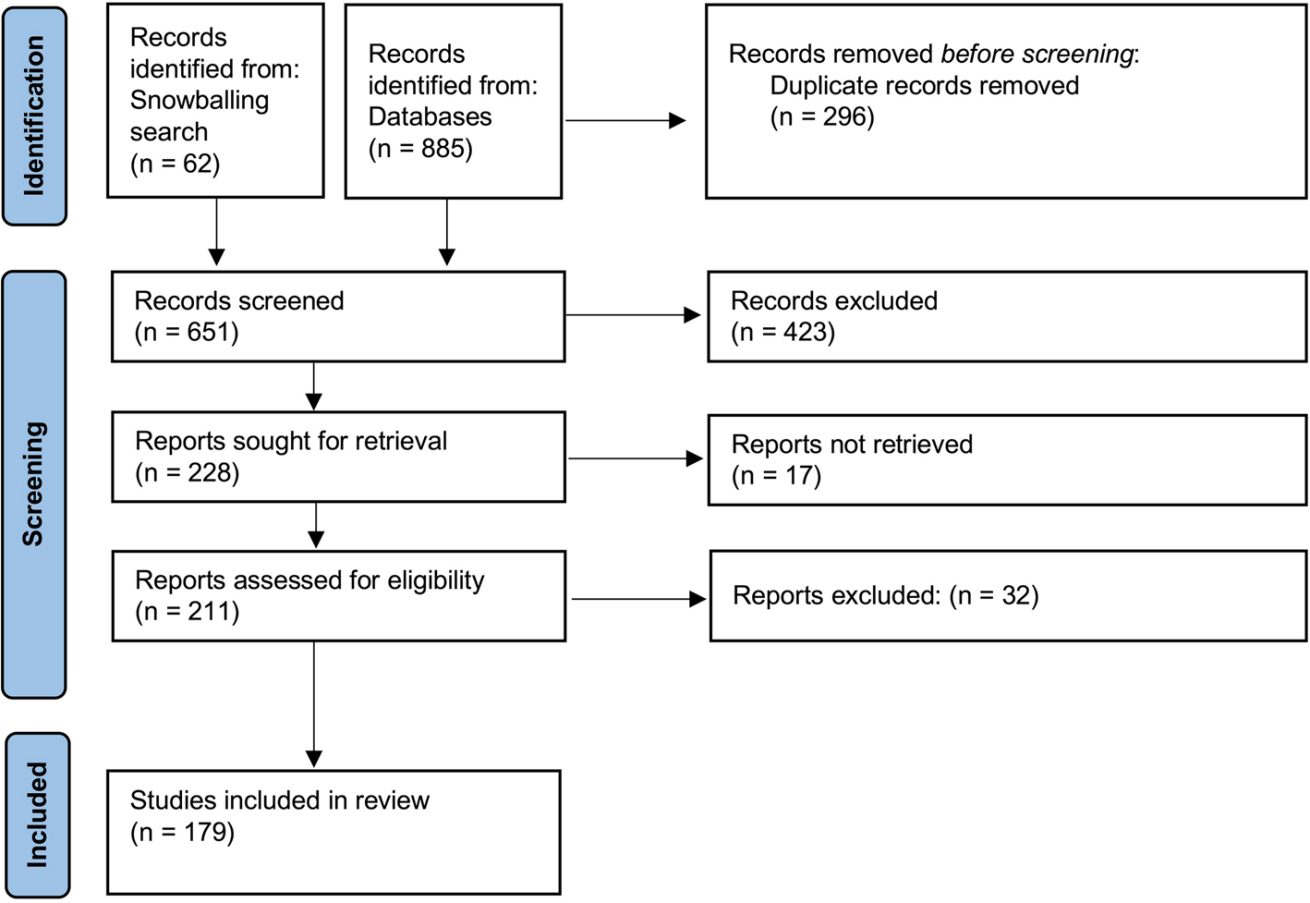
Flowchart of the systematic search and selection process following the Prisma statement

A comprehensive literature search of the medical databases Web of Science, Embase and PubMed was set up. The search was conducted on March 27, 2021. An overview of the search strategy is attached as Addendum 1. The data selection was conducted by two reviewers (PPS and AM) according to the Preferred Reporting Items for Systematic Reviews and Meta-analyses (PRISMA) guidelines. (Fig. 2) First, duplicates were eliminated. Second, titles and abstracts were screened for relevance. Afterwards, full-text articles were assessed for eligibility. One reviewer (AM) conducted a snowballing search to avoid missing any relevant articles. Table 1 shows a detailed overview of the inclusion and exclusion criteria.

**Table 1:** Detailed inclusion and exclusion criteria

Inclusion criteria	Exclusion criteria
- Original publications in English language with full-text available	- Not related to defined outcomes of interest (Ocular adverse events)
- Assessed occurrence of ocular adverse events	- Not related to immune checkpoint inhibitors
- Included cancer patients treated with (a combination of) immune checkpoint inhibitors	*- *In vitro/not in humans
- Comprehensive description of the case report	- Language other than English

## Results

One hundred seventy-nine studies were included, containing a total of 290 cases of ocular adverse events associated with ICIs. An overview of these case reports is attached as Addendum 2. In 179 of these cases the primary tumor was melanoma (61.7%). Lung cancer was the primary tumor in 56 cases (19.3%), including 6 cases of small-cell lung cancer. Other primary tumors were urological tumors (*n* = 28; 9.7%), gynecological tumors (*n* = 6; 2.1%) and gastro-intestinal cancer (*n* = 5; 1,7%). The other cases described glioblastoma, Hodgkin lymphoma, leukemia, hypopharyngeal cancer, leiomyosarcoma, Merkel cell carcinoma, parotid cancer, squamous cell carcinoma and thymic cancer as primary tumors.

**Fig. 3:**
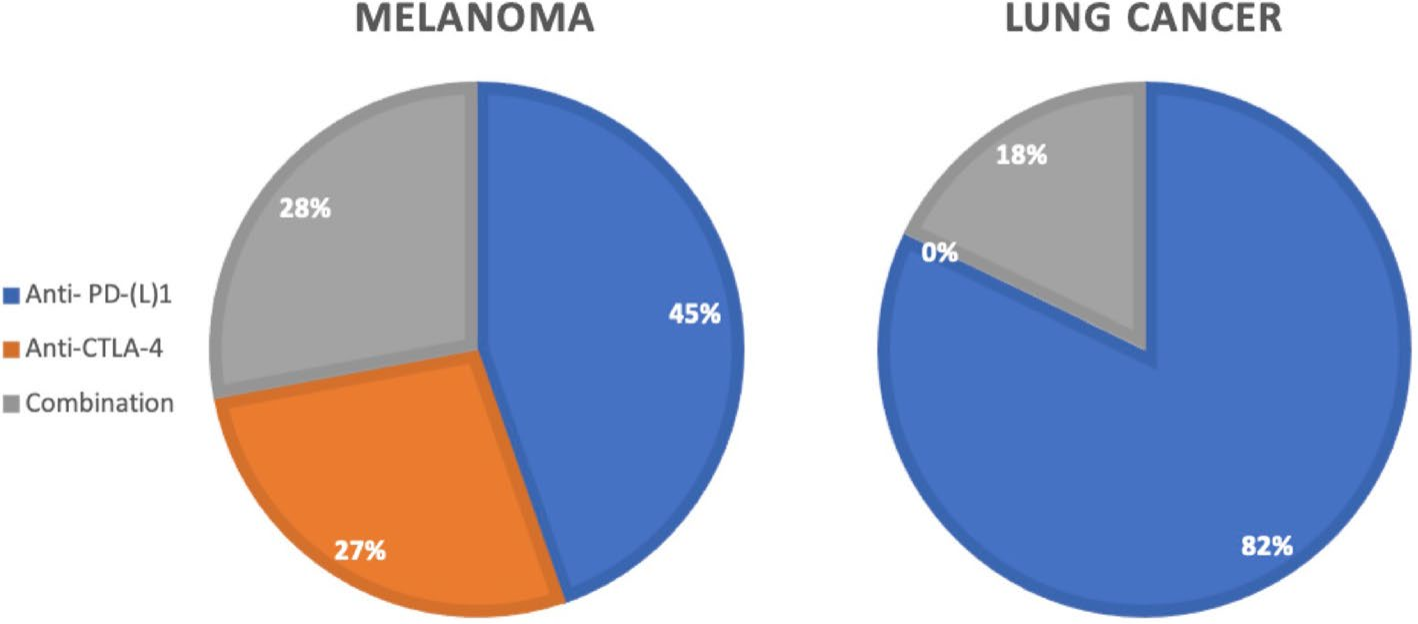
Use of ICIs related to the primary tumor

The different ICIs used are depicted in Table 2. In 68 cases a combination of anti-PD-(L)1 and anti-CTLA-4 products was used (23.4%). The use of the diverse types of ICIs is related to the type of the primary cancer (Fig. 3).

**Table 2:** Use of ICIs

	Total = single use + combination therapy N (%)	Single use N (%)
Anti-PD-1		148 (51.0%)
**Pembrolizumab**	93 (32.1%)	81
**Nivolumab**	123 (42.5%)	65
**Cemiplimab**	1 (0.3%)	1
**Sintilimab**	1 (0.3%)	1
Anti-PD-L1		22 (7.6%)
**Atezolizumab**	10 (3.4%)	10
**Avelumab**	4 (1.4%)	4
**Durvalumab**	8 (%)	6
Anti-CTLA-4		52 (17.9%)
**Ipilimumab**	116 (40.0%)	50
**Tremelimumab**	3 (1.0%)	1

**Fig. 4:**
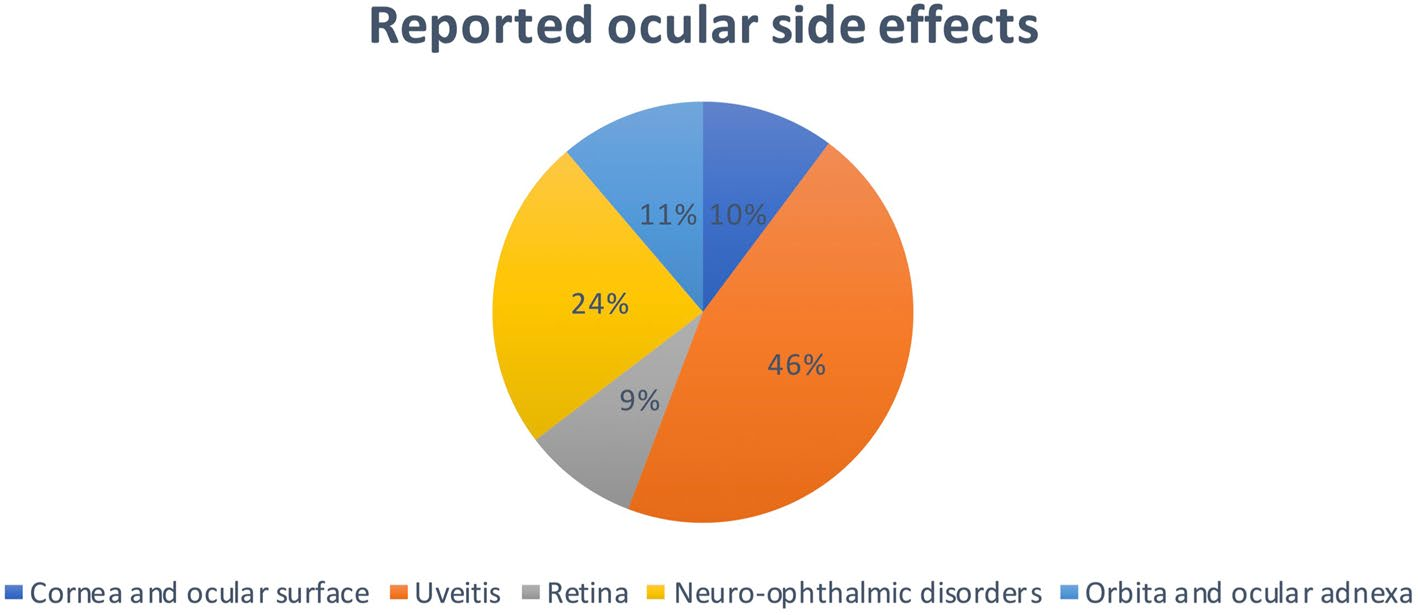
Reported ocular side effects

**Fig. 5:**
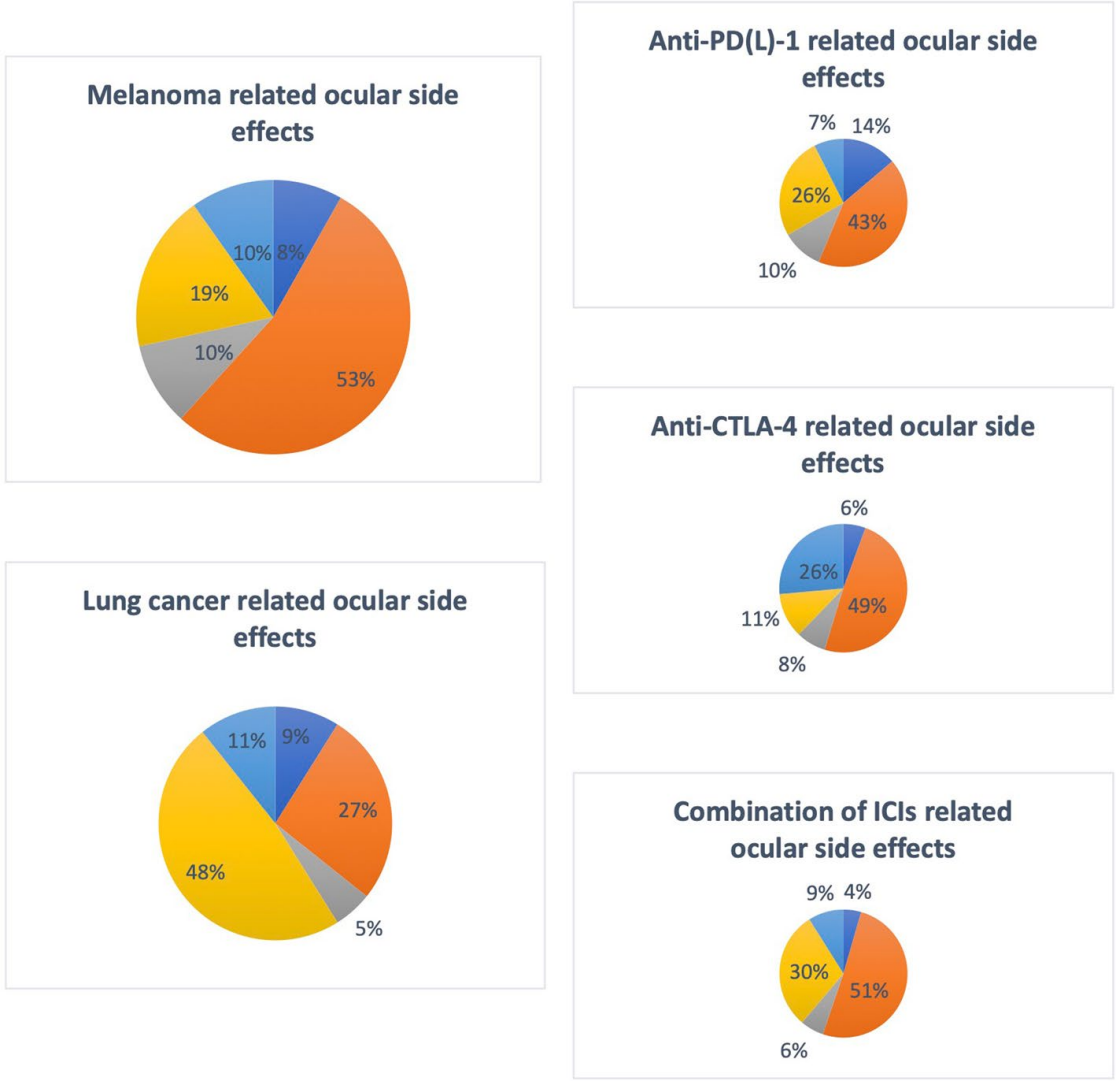
Reported ocular side effects according to the primary tumor / ICI used

The most common reported ocular side effects were uveitis (*n* = 134) and neuro-ophthalmic disorders (*n* = 71). Additionally, we reported side effects involving the cornea and ocular surface (*n* = 30), retina (*n* = 26) and orbit (*n* = 33) (Fig. 4). Table 3 illustrates demographic characteristics of these groups. The maximum range in time to onset of different adverse events from initiation of ICI therapy is depicted in Table 4. Figure 5 and Table 5 show the reported ocular side effects according to the primary tumor or ICI used.

**Table 3:** Demographic characteristics

	**Cornea and ocular surface**	**Uveitis**	**Retina**	**Neuro-ophthalmic disorders**	**Orbit and ocular adnexa**
Mean age	58.7	59.8	60.8	65.8	64.6
M/F ratio	17/13	70/62	13/12	47/23	21/11
Race (if reported)					
- Caucasian	4	39	5	9	5
- Asian		10	2	1	
- Afro-American		7		1

**Table 4:** Time to onset of the different adverse events

Shortest Time to Event (after initiation of treatment)	Longest Time to Event (after initiation of treatment)
**Uveitis**	
2 weeks	2 years
**Neuro-ophthalmic disorders**	
1 week	5 years
**Orbit and ocular adnexa**	
10 days	8 months
**Retina**	
5 days	2 months
**Cornea and ocular surface**	
1 week	18 months

**Table 5:** Overview of reported ocular side effects related to primary tumor and ICI used

	Total N (%)	Melanoma N (%)	Lung cancer N (%)	Anti-PD-(L)1 N (%)	Anti-CTLA4 N (%)	Combination N (%)
**Uveitis** [8–85]	**134 (46.2)**	**98 (54.7)**	**15 (26.8)**	**74 (43.5)**	**26 (50.0)**	**34 (50.0)**
Anterior uveitis	60 (20.7)	38 (21.2)	6 (10.7)	13 (7.6)	12 (23.1)	15 (22.1)
Intermediate uveitis	8 (2.8)	6 (3.4)	1 (1.8)	3 (1.8)	3 (5.8)	2 (2.9)
Posterior uveitis	5 (1.7)	3 (1.7)		3 (1.8)	2 (3.8)	
Panuveitis	26 (9.0)	18 (10.1)	4 (7.1)	12 (7.1)	3 (5.8)	4 (4.4)
VKH like uveitis	30 (10.3)	26 (14.5)	3 (5.4)	17 (10.0)	5 (9.6)	8 (11.8)
Birdshot like uveitis	1 (0.3)	1 (0.6)		1 (0.6)		
Undifferentiated uveitis	5 (1.7)	3 (1.7)	1 (1.8)	3 (1.8)	2 (3.8)	
Sarcoidosis	6 (2.1)	4 (2.2)	1 (1.8)	5 (2.9)		1 (1.5)
**Neuro-ophthalmic complications** [8–10, 22, 32, 59, 86–133]	**71 (24.5)**	**34 (19.0)**	**27 (48.2)**	**45 (26.5)**	**6 (11.5)**	**20 (29.4)**
Myasthenia gravis	36 (12.4)	16 (8.9)	16 (28.6)	27 (15.9)	3 (5.8)	6 (8.8)
Optic Nerve disorders	27 (9.3)	15 (8.4)	8 (14.3)	13 (7.6)	3 (5.8)	11 (16.2)
Other Cranial Nerve disorders	6 (2.1)	3 (1.7)	1 (1.8)	3 (1.8)		3 (4.4)
LEMS	2 (0.7)		2 (3.6)	2 (1.2)		
**Orbit and ocular adnexa** [11, 14, 134–158]	**33 (11.4)**	**18 (10.1)**	**6 (10.7)**	**13 (7.6)**	**14 (26.9)**	**6 (8.8)**
Myositis/Myopathy	12 (4.1)	4 (2.2)	4 (7.1)	8 (4.7)	1 (1.9)	3 (4.4)
Graves' disease/Thyroid (like) eye disease	10 (3.4)	5 (2.8)	2 (3.6)	3 (1.8)	4 (7.7)	3 (4.4)
Orbitopathy	10 (3.4)	9 (5.0)		2 (1.2)	8 (15.4)	
Lacrimal gland	1 (0.3)				1 (1.9)	
**Cornea and ocular surface** [9–15, 159–170]	**30 (10.3)**	**15 (8.4)**	**5 (8.9)**	**24 (14.1)**	**3 (5.8)**	**3 (4.4)**
Conjunctivitis	5 (1.7)	2 (1.1)	2 (3.6)	3 (1.8)	1 (1.9)	1 (1.5)
Keratitis	3 (1.0)	3 (1.7)		1 (0.6)	2 (3.8)	
(Epi)scleritis	1 (0.3)			1 (0.6)		
Dry eye / Sicca	14 (4.8)	8 (4.5)	1 (1.8)	12 (7.1)	1 (1.9)	1 (1.5)
Corneal toxicity	7 (2.4)	4 (2.2)	1 (1.8)	6 (3.5)		1 (1.5)
Corneal graft rejection	2 (0.7)		1 (1.8)	2 (1.2)		
**Retina** [9, 12, 23, 134, 171–186]	**26 (9.0)**	**18 (10.1)**	**3 (5.4)**	**18 (10.6)**	**4 (7.7)**	**4 (5.9)**
MAR	7 (2.4)	7 (3.9)		3 (1.8)	1 (1.9)	3 (4.4)
AEPVM	7 (2.4)	7 (3.9)		5 (2.9)	2 (3.8)	
Autoimmune retinopathy	2 (0.7)			2 (1.2)		
AMN	6 (2.1)		1 (1.8)	6 (3.5)		
Fundus depigmentation	3 (1.0)	3 (1.7)	2 (3.6)	2 (1.2)		1 (1.5)
CNV	1 (0.3)	1 (0.6)			1 (1.9)	

*VKH* Vogt-Koyanagi-Harada, *LEMS* Lambert Eaton Myasthenic Syndrome, *MAR* Melanoma Associated Retinopathy, *AEPVM* Acute Exudative Vitelliform Maculopathy, *AMN* Acute Macular Neuroretinopathy, *CNV* Choroidal NeoVascularization

### Uveitis

Uveitis is frequently reported as ocular adverse event related to ICIs. Besides anterior uveitis, Vogt-Koyanagi-Harada (VKH) like disease is an important adverse event in this group. Out of the 31 reported VKH like uveitis cases, only a few authors mentioned ethnical background: 8 patients were Asian, and 5 patients were Caucasian. Overall, uveitis is mostly associated with melanoma.

### Neuro-ophthalmic disorders

Myasthenia gravis (MG) is the most reported neuro-ophthalmic disorder (Fig. 5). In general, neuro-ophthalmic disorders are more prevalent in lung cancer compared to melanoma patients (Fig. 5).

### Orbit and ocular adnexa

An overview of all reported adverse events involving the orbit and ocular adnexa is depicted in Table 5.

### Cornea and ocular surface

Dry eye disease (DED) and corneal toxicity were the primary reported adverse events involving the cornea and ocular surface (Table 5).

### Retina

In 18 melanoma patients, a retinal side effect was reported. All the melanoma associated retinopathy (MAR), acute exudative vitelliform maculopathy (AEPVM), choroidal neovascularization (CNV) and fundus depigmentation cases are related to melanoma. Two cases of acute macular neuroretinopathy (AMN) and one case of autoimmune retinopathy *are related to lung cancer *(Table 5)*.*

### Management

Overall, the treatment of the different adverse events included administration of steroids (topical, intravitreal or systemic), with or without the discontinuation of the ICI. A detailed description of the treatment in each case can be found in Addendum 2. Table 6 shows the results of steroid administration. Table 7 shows the number of cases in which the ICI was discontinued.

**Table 6:** Results of steroid administration

	**Cases steroids used**	**Resolution**	**Partial resolution**
Uveitis (*n* = 134)	124 (92.5%)	82/124 (66.1%)	20/124 (16.1%)
Neuro-ophthalmic disordes (*n* = 71)	67 (94.3%)	26/67 (38.3%)	20/67 (29.9%)
Orbit and ocular adnexa (*n* = 33)	31 (93.9%)	20/31 (64.5%)	7/31 (22.6%)
Cornea and ocular adnexa (*n* = 30)	25 (83.3%)	13/25 (52.0%)	9/25 (36.0%)
Retina (*n* = 26)	13 (50.0%)	4/13 (30.8%)	3/13 (23.1%)

**Table 7:** ICI discontinuation

	**Cases ICI discontinued**
Uveitis (*n* = 134)	79 (59.0%)
Neuro-ophthalmic disordes (*n* = 71)	53 (74.6%)
Orbit and ocular adnexa (*n* = 33)	23 (69.7%)
Cornea and ocular adnexa (*n* = 30)	20 (66.7%)
Retina (*n* = 26)	16 (61.5%)

## Discussion

This review shows that ocular adverse events related to the use of ICIs vary widely. We suggest that most adverse events related to ICIs can be divided into two distinct groups.

The first group consists of immune-related adverse events (irAEs). In this group, inhibition of checkpoints, leads to stimulation of T-cells that can attack not only tumor cells, but also normal cells. This leads to an auto-immune response that can affect any organ, including the eyes. Immune related adverse events are thought to be caused by a direct effect of ICIs, leading to a stimulated off-target cellular immune response [4, 187]. Theoretically, this immune response wanes again after stopping the ICIs. In practice however, corticosteroids or corticosteroid sparing immunosuppressive treatment might be necessary, especially to control severe irAEs [188].

The second group of adverse events might be due to induction or exacerbation of paraneoplastic syndromes triggered using ICIs. Paraneoplastic syndromes can be defined as remote effects of cancer that are not caused by the tumor and its metastasis. Paraneoplastic syndromes are thought to be due to an immune response against the tumor with cross-reaction of antibodies between tumor- and self-antigens [189, 190]. In this group, ICIs may contribute to autoimmunity, which may involve increased production of cross-reactive antibodies. By stimulating the anti-tumoral immune response, the cross-reactive immune response is increased, which can lead to aggravation of the paraneoplastic syndrome [191]. Treatment of paraneoplastic syndromes is particularly challenging. On the one hand, immunosuppressive treatment can decrease auto-immunity, but might have a detrimental effect on tumor progression. On the other hand, eventually paraneoplastic syndromes can fade when the tumor is controlled. If the tumor is completely suppressed, the anti-tumor immune response decreases, as does the cross-reactive immune response. In contrast to irAEs, paraneoplastic syndromes are related to the tumor status.

In addition, the time to onset varies widely, with a range between 5 days and 5 years after initiation of treatment. A precise determination of the mean time to onset was impossible to compute given the lack of consistent reporting. However, a detailed description of the time to onset of the adverse event in each case is attached in Addendum 2. This review shows that a clinician should always be aware of possible ocular adverse events, at any point during and after the treatment with ICIs.

### Uveitis

Previous studies have already described a possible link between VKH disease and cutaneous malignant melanoma. The hypothesis is that an immune response against melanoma also attacks normal melanin containing tissue, leading to VKH disease [192]. When stimulating the immune system by blocking its checkpoints, not only the reaction against melanoma cells strengthens, but also the reaction against melanin-containing normal tissue. This might explain why VKH like uveitis is relatively common in melanoma patients treated with ICIs. Several studies have shown an association between VKH disease and particular HLA-types such as HLA-DR4 [65, 192, 193]. In the future, HLA typing might contribute to the selection of patients that are at risk to develop VKH like uveitis with ICIs.

In literature, one case with ocular features of Birdshot uveitis (BU) is described, related to treatment with ICIs for melanoma [81] BU is an auto-immune disease characterized by bilateral chronic posterior uveitis with stromal choroiditis and retinal vasculitis. This condition is most seen in middle-aged Caucasian patients [194]. An extraordinarily strong association with HLA-A29 has been described. Over 95%, if not all the patients with BU are HLA-A29 positive [81]. Nevertheless, in the case described by Acaba-Berrocal et al. the patient tested negative for HLA-A29. The authors hypothesize that pembrolizumab induced auto-immunity and thereby caused Birdshot-like uveitis.

In summary, the frequent occurrence of VKH like uveitis and the description of a case of Birdshot like uveitis in patients treated with ICIs, supports the theory of T-cell immunity against melanocytes in these diseases. Furthermore, it might reflect that these diseases might be caused by an oncogenic trigger that leads to an anti-melanocytic immune response in patients that are genetically predisposed.

### Neuro-ophthalmic disorders

Both MG exacerbations and new onset MG have been found to occur with the use of ICIs [91]. A recent review shows that MG is the most common neuromuscular adverse event related to the use of ICIs [195]. However, MG occurs predominantly in anti-PD-1 therapy, but rarely with anti-CTLA-4 monotherapy [89]. Findings in our review confirm this assumption. Out of 28 cases, 21 are related to anti-PD-(L)-1, 3 to anti-CTLA-4 and 4 to a combination treatment. However, lung cancer is mostly treated with anti-PD(L)-1 and less frequently with anti-CTLA-4 monotherapy [196]. As neuro-ophthalmic disorders are mostly linked to lung cancer; this could explain why it is less common with anti-CTLA-4 monotherapy.

In many reports, MG is thought to be an irAE. T lymphocytes play a role in the pathogenesis [94, 107]. However, another hypothesis states that MG is a paraneoplastic syndrome, mainly associated with lung cancer. MG is frequently associated with non-small cell lung cancer, but it has been described in small cell lung cancer as well [197].

Optic nerve disorders, including optic neuritis (ON) and optic atrophy, are reported as well. ON associated with ICIs seems to have a unique presentation compared with typical ON. Its presentation is more often bilateral with a painless reduction of visual acuity, while color vision remains very often intact. Moreover, some patients do not experience vision recovery while visual acuity normally improves within 4 weeks in typical ON [121, 127]. Vogrig et al. suggest that a demyelinating process could be relevant in ON triggered by ICIs. They speculate that the pathogenesis is immune-mediated. Several observations, such as inflammatory alterations in CSF and a good response to corticosteroids support this hypothesis [127]. However, ON has been described as a paraneoplastic syndrome related to lung, breast, genitourinary, nasopharyngeal, thyroid cancers, and thymoma [198]. This might indicate that ICI induced ON is a paraneoplastic phenomenon, exacerbated by ICIs.

### Orbit and ocular adnexa

Orbital myositis as well as orbitopathy resembling thyroid eye disease (TED) have been associated with ICIs [147]. In this review, 11 cases of myositis/myopathy were identified. We believe that in some of these cases myositis and MG might co-exist. Overlapping myositis with myasthenia has been reported in approximately 5% of the generalized myositis cases [137]. In addition, a review by Bitton et al. reported a series of patients treated with anti-PD-(L)1 who experienced concomitant myositis and MG [11]. Garibaldi et al. suggest a common pathogenic pathway in ICI-related myositis and thymoma MG, since anti-striatal antibodies are found in both [137]. These myositis cases show great resemblance with the reported cases of inflammatory orbitopathy suggesting it involves the same clinical entity. Clinical features are different from TED-like eye disease (e.g., tendons are involved in muscle enlargement, lid lag is absent) [153, 156].

Graves’ disease and TED-like eye disease have been reported in 10 cases, included in this review. The etiology of Graves’ disease is known to be multifactorial, consisting of genetic predisposition, environmental factors, and stress and other issues. Several genes have been identified to play a plausible role in the generation of orbital inflammation, including CTLA-4 gene polymorphisms. These polymorphisms may translate an ineffective receptor which leads to inadequate suppression of the immune system with T-lymphocyte activation and proliferation as a result [145]. However, multiple studies, including meta-analyses, have been inconsistent in showing a significant association between thyroid orbitopathy and CTLA-4 gene polymorphisms [147, 148]. The fact that anti-CTLA-4 treatment provokes Graves’ disease might revive this hypothesis [135, 150].

### Cornea and ocular surface

Dry eye disease (DED) is the primary reported adverse event involving the ocular surface. However, we think this is an underestimation of the prevalence of DED. Very often, DED is not severe and is easily solved with artificial tears, hence it seems less important to report. A systematic review showed that the incidence of DED related to the use of ICIs ranges from 1.2% to 24.2% [199] The fact that these values vary widely, is consistent with our hypothesis that DED is not always reported consistently. Nevertheless, the impact on the quality of life of the patient can be important and severe DED can eventually progress to corneal perforation [159, 168].

Another adverse event involving the ocular surface is corneal graft rejection, reported in two cases. Due to the generalized stimulation of the cellular immune system, graft rejection may be enhanced. These cases show the relevance of considering all the risks including corneal graft rejection, compared to the benefits before starting ICI treatment in selected patients.

### Retina

Melanoma associated retinopathy (MAR), carcinoma associated retinopathy (CAR) and paraneoplastic acute exudative vitelliform maculopathy (pAEPVM) are all known paraneoplastic ocular syndromes. As the name indicates, MAR is exclusively related to melanoma. MAR is a subclass of auto-immune retinopathy in which autoantibodies target certain melanoma antigens. These activated autoantibodies then cross-react mostly with retinal bipolar cells and cause retinopathy [200]. By initiating treatment with ICIs and unleashing our immune system, MAR can be initiated or exacerbated. This has been described in multiple cases [9, 12, 23, 171–174]. However, Khaddour et al. describe a case of MAR resolution after initiation of pembrolizumab [201]. They suggest that ICIs can either stimulate or resolve paraneoplastic syndromes. The possible underlying mechanism is that increased T-cell activity leads to shrinkage of the primary tumor, by which the reactive cross-reactive antibodies also diminish.

The pathogenesis of CAR is similar to that of MAR. CAR is most often seen in patients with lung cancer but can be found in multiple tumors [202]. In contrast to MAR, the immune reaction is targeted to the photoreceptors in most cases, leading to rapid irreversible visual loss. In this review, we found one case of CAR after nivolumab treatment for NSCLC. Similar to MAR, visual prognosis is often very poor [181]. AEPVM is a disease characterized by subretinal accumulation of hyperautofluorescent yellow subretinal deposits in the posterior pole and multifocal areas of vitelliform (egg yolk-like) serous detachments. The underlying pathophysiology is not fully understood. The mechanism of pAEPVM is probably based on an immune reaction against retinal pigment epithelium (RPE) cells. It has been documented in several melanoma and carcinoma cases [179]. Remarkably, in this review AEPVM was related to mucosal melanoma in 4 out of 7 cases.

In 3 cases, fundus depigmentation without signs of ocular inflammation, was documented after ICI treatment. There might be a link with integumentary changes (e.g., vitiligo) which are previously documented after ICI treatment [203]. In 2 cases choroidal nevi regression was described as well. These findings seem to be induced by an immune-mediated reaction against self-melanocytes, like in VKH like uveitis. But unlike VKH disease, there is no inflammation of the eye [182–184].

Acute macular neuroretinopathy (AMN) is a rare disease characterized by a sudden decrease of central visual acuity and central scotomata. The clinical picture consists of reddish-brown, wedge-shaped lesions surrounding the fovea. Patients are most often female and quite young (mean age at presentation 29.5 years old). Multiple factors are identified as triggers for AMN, including fever or flu-like illness and oral contraceptives. AMN is thought to result from ischemia in the outer capillary layer of the retina [204]. Remarkably, all the reported cases of AMN in this study are related to the use of atezolizumab. The mean age of the patients is 43 years old, which is young. In all these cases the patients reported fever or flu-like symptoms and acute vision loss within 2 weeks after atezolizumab initiation. A T-cell mediated response could be a trigger for AMN since in all these cases PD-L1 was blocked. However, we think AMN might be triggered by fever, as described earlier. Fever and flu-like symptoms are the primary reported side effects in anti-PD-L1 clinical trials [186] This might be consistent with the hypothesis that AMN is a result of oxidative stress, triggered by fever, which is related to the use of atezolizumab [205].

### Management

There are no clear guidelines on how to prevent and treat ocular adverse events related to the use of ICIs. A baseline ophthalmologic examination is recommended before starting treatment with anticancer agents that can induce ocular adverse events. In addition, clinicians should be aware of these ocular adverse events as swift referral to an ophthalmologist could be crucial. Overall, the management of ocular adverse events related to the use of ICIs depends on the severity of the adverse event [206]. Hereby, it is best to strive for local treatment and continuation of the ICI. In all cases included the first step in treatment was corticosteroid administration. These can be administered locally (eyedrops, periocular or intraocular injections) or systemic (oral or intravenous). The effect of corticosteroids on the efficacy of immunotherapy is still not clear [206]. However, chronic corticosteroid treatment is associated with harmful effects, implicating it might be better to avoid systemic corticosteroids if possible [207]. Additionally, adverse-event specific therapy might be associated, such as artificial tears and topical cyclosporine for DED and acetylcholinesterase inhibitors for MG. In case of severe adverse events, it might be indicated to stop treatment with ICIs. In this review, in 179 out of 290 cases the ICI was discontinued. As mentioned before, if the adverse event is a paraneoplastic event, it might be better to continue ICI treatment to achieve tumor control. However, some cases described worsening of paraneoplastic events during ICI treatment [208]. This might be due to the stimulation of an underlying T-cell pathway by the ICI. In practice, it might be better to discontinue the ICI treatment if it is uncertain if the paraneoplastic event will fade.

We believe most cases of corneal and ocular surface disease can be treated locally. The treatment of uveitis depends on the severity. In case of anterior uveitis, treatment can be local. In severe VKH like uveitis, corticosteroids are often needed to resolve the serous retinal detachments. The question remains if ICIs must be stopped. In MAR and CAR, the sudden and dramatic loss of vision makes it difficult to make a therapeutic decision. ICIs are stopped mostly, and corticosteroids are started, with variable results. Unfortunately, there is no proven treatment in these cases and the visual prognosis is poor.

Unlike MAR and CAR, pAEPVM is known to be reversible and the visual prognosis is good. The reported cases are conflicting. It is not clear if discontinuation of the ICIs leads to resolution of the symptoms or anatomical changes. However, stopping ICIs can have a negative impact on overall survival. Because the anatomic changes are reversible and because the visual prognosis is favorable, the most important goal in these cases is to strive for tumor control, which may involve continuation of ICIs. Due to better tumor control, the immune reaction against the tumor can diminish, and thereby the immune response against the RPE. This can ultimately lead to resolution of the vitelliform material with preserved visual acuity [175].

In case of severe neurological ocular side effects, corticosteroids are warranted.

There is an unmet medical need to treat the most severe ocular adverse events, without interfering with the effectivity of the ICI. Numerous further immunotherapy approaches are in more experimental phases of the pipeline, such as inactivation of autoantibodies with bacterial enzymes that either cleave or enzymatically deglycosylate immunoglobulins [209, 210]. Also, inhibition of the neonatal Fc receptor (FcRn) with efgartigimod or rozanolixizumab reduced IgG concentrations in phase II trials in patients with myasthenia gravis [211, 212]. Hopefully, these novel options can open new therapeutic avenues in these challenging cases. 

### Limitations

We think this review has an important impact on the current knowledge and management of ocular adverse events related to the use of ICIs. However, there are limitations since only case reports were included. First, our results might be an underestimation due to lack of voluntary reporting. We suggest that mostly mild adverse events, such as DED, would be missed. Second, only correlations can be described. Further research will be needed to confirm a causal relationship between a certain ICI, tumor or risk factor and an adverse event. However, to our knowledge, this study is the most comprehensive review of ocular adverse events related to the various cancers and different ICIs.

## Future perspectives

In the future, risk assessment before starting ICIs might contribute to reduce or to diagnose adverse events earlier. We assume that the type of adverse event is related to the underlying primary tumor, rather than to the ICI used. Uveitis, MAR and AEPVM are mostly associated with melanoma. While CAR, neuro-ophthalmic disorders such as MG and ON and orbital disorders are most often related to lung cancer. An exception to this finding, is AMN which is closely related to the use of atezolizumab and fever and orbitopathy which is linked to anti-CTLA-4 treatment. Certain genetic predispositions have already been identified, such as HLA-DR4 in VKH like uveitis. It seems important to further characterize these clinical entities to identify patients at risk of developing ocular adverse events. The aim of this paper was to provide an overview of all reported ocular adverse events related to the use of ICIs. Similar reviews of adverse events of ICIs have been published. However, we believe our review has an added value in comparing ocular adverse events in different types of tumors and with different ICI types. The insights retrieved from this review might contribute to a better understanding of the underlying mechanisms of these ocular adverse events and might shed new lights on the pathophysiology of different eye diseases. Particularly, the difference between actual irAEs and paraneoplastic syndromes might be relevant. We believe these hypotheses are of great value compared to previous articles. Further research is needed to investigate these underlying mechanisms, possible genetic predispositions, and other risk factors, to provide a patient-centered care.

## Supplementary Information

**Additional file 1.** 

**Additional file 2.**

## Data Availability

The dataset supporting the conclusions of this article is included within the article and its additional file(s).

## Abbreviations

AEPVM: Acute exudative vitelliform maculopathy
AMN: Acute macular neuroretinopathy
BU: Birdshot uveitis
CNV: Choroidal neovascularization
CTLA-4: Cytotoxic T-lymphocyte-associated antigen 4
DED: Dry eye disease
FcRn: Neonatal Fc receptor
ICIs: Immune checkpoint inhibitors
IgG: Immunoglobulin G
irAEs: Immune-related adverse events
LEMS: Lambert Eaton Myasthenic Syndrome
MAR: Melanoma associated retinopathy
MG: Myasthenia gravis
NSCLC: Non-small cell lung cancer
ON: Optic neuritis
PD-1: Programmed cell death protein 1
PD-L1: Programmed death-ligand 1
PRISMA: Preferred Reporting Items for Systematic Reviews and Meta-analyses
TED: Thyroid eye disease
VKH: Vogt-Koyanagi-Harada
RPE: Retinal pigment epithelium
