# Supplementary material for: Ocular adverse events associated with immune checkpoint inhibitors, a scoping review
Source: J Ophthalmic Inflamm Infect. 2023 Feb 22;13:5. doi: 10.1186/s12348-022-00321-2 (PMC9947214; doi:10.1186/s12348-022-00321-2)
Supplement: Supplementary file 1 — Additional file 1. [file 12348_2022_321_MOESM1_ESM.pdf]

Search strategy

Pubmed

- Concept 1: Immune checkpoint inhibitors

Checkpoint-inhibit*[tiab] OR checkpoint-block*[tiab] OR "Programmed Cell Death 1 Receptor/antagonists and inhibitors"[Mesh] OR "CTLA-4 Antigen/antagonists and inhibitors"[Mesh] OR ipilimumab[tiab] OR “ipilimumab”[MeSH] OR nivolumab[tiab] OR “nivolumab”[MeSH] OR pembrolizumab[tiab] OR “pembrolizumab”[supplementary concept] OR cemiplimab[tiab] OR “cemiplimab”[supplementary concept] OR atezolizumab[tiab] OR “atezolizumab”[supplementary concept] OR avelumab[tiab] OR “avelumab”[supplementary concept] OR durvalumab[tiab] OR “durvalumab”[supplementary concept] OR spartalizumab OR tremelimumab[tiab] OR ticilimumab[tiab] OR "tremelimumab"[supplementary Concept] OR camrelizumab [tiab] OR sintilimab [tiab] OR tislelizumab[tiab] OR toripalimab[tiab] OR dostarlimab[tiab]

- Concept 2: Ophthalmologic

“Eye”[MeSH] OR eye*[tiab] OR ophthalm*[tiab] OR ocular[tiab] OR oculomotor[tiab] OR ocular-adnexa[tiab] OR cornea*[tiab] OR uvea*[tiab] OR retina*[tiab] OR conjunctiva*[tiab] OR iris[tiab] OR ciliary-body[tiab] OR trabecular-meshwork[tiab] OR choroid*[tiab]OR lens[tiab] OR optic[tiab] OR orbit*[tiab] OR vitreous[tiab] OR vitreum[tiab] OR vitreal[tiab] OR sclera*[tiab] OR extraocular-muscle*[tiab] OR intraocular-pressure[tiab] OR lacrimal[tiab]

- Concept 3: Adverse events

“Drug-related side effects and adverse reactions”[Mesh] OR adverse-effect*[tiab] OR adverse-reaction*[tiab] OR side-effect*[tiab] OR complication*[tiab] OR toxic*[tiab] OR “Eye diseases”[Mesh] OR orbitopath*[tiab] OR ophthalmopath*[tiab] OR inflammat*[tiab] OR cellulitis[tiab] OR Graves[tiab] OR thyroid-eye-disease[tiab] OR tolosa-hunt[tiab] OR myopath*[tiab] OR myositis[tiab] OR palsy[tiab] OR palsies[tiab] OR paralysis[tiab] OR myoplegia[tiab] OR ophthalmoplegia[tiab] OR myasthenia[tiab] OR "Myasthenia Gravis"[Mesh] OR uveitis[tiab] OR panuveitis[tiab] OR planitis[tiab] OR vitritis[tiab] OR iritis[tiab] OR keratouveitis[tiab] OR iridocyclitis[tiab] OR white-dot-syndrome*[tiab] OR episcleritis[tiab] OR scleritis[tiab] OR conjunctivitis[tiab] OR dry-eye*[tiab] OR sicca[tiab] OR Sjogren*[tiab] OR "Sjogren's Syndrome"[Mesh] OR retinitis[tiab] OR chorioretinitis[tiab] OR vitreoretinopath*[tiab] OR chorioretinopath*[tiab] OR retinopath*[tiab] OR maculopath*[tiab] OR drusen*[tiab] OR detachment[tiab] OR neovascularization[tiab] OR vasculopath*[tiab] OR epiretinal-membrane*[tiab] OR vasculitis[tiab] OR arteritis[tiab] OR arteriitis[tiab] OR "Giant Cell Arteritis"[Mesh] OR necrosis[tiab] OR hemorrhag*[tiab] OR occlusion[tiab] OR neuroretinitis[tiab] OR neuroretinopath*[tiab] OR choroiditis[tiab] OR effusion*[tiab] OR choroideremia[tiab] OR choroidopath*[tiab] OR sarcoidosis[tiab] OR "Sarcoidosis"[Mesh] OR papillitis[tiab] OR papilledema[tiab] OR edema[tiab] OR glaucoma[tiab] OR neuropath*[tiab] OR neuritis[tiab] OR atrophy[tiab] OR neuromyelitis[tiab] OR nystagmus[tiab] OR strabismus[tiab] OR keratoconus[tiab] OR keratitis[tiab] OR ulcer*[tiab] OR infectio*[tiab] OR

epitheliopath*[tiab] OR endophthalmitis[tiab] OR graft-rejection[tiab] OR "Graft Rejection"[Mesh] OR hypotension[tiab] OR hypertension[tiab] OR cataract[tiab] OR opacit*[tiab] OR dacryocystitis[tiab]

Embase

- Concept 1: Immune checkpoint inhibitors

‘Checkpoint inhibit*’:ti,ab,kw OR ‘checkpoint block*’:ti,ab,kw OR 'programmed death 1 receptor inhibitor'/exp OR 'cytotoxic t lymphocyte antigen 4 inhibitor'/exp OR ‘ipilimumab’:ti,ab,kw OR 'ipilimumab'/exp OR ‘nivolumab’:ti,ab,kw OR 'nivolumab'/exp OR ‘pembrolizumab’:ti,ab,kw OR 'pembrolizumab'/exp OR ‘cemiplimab’:ti,ab,kw OR 'cemiplimab'/exp OR ‘atezolizumab’:ti,ab,kw OR 'atezolizumab'/exp OR ‘avelumab’:ti,ab,kw OR 'atezolizumab'/exp OR ‘durvalumab’:ti,ab,kw OR 'durvalumab'/exp OR ‘tremelimumab’:ti,ab,kw OR ‘ticilimumab’:ti,ab,kw OR 'ticilimumab'/exp OR ‘spartalizumab’:ti,ab,kw OR ‘camrelizumab’:ti,ab,kw OR ‘sintilimab’:ti,ab,kw OR ‘tislelizumab’:ti,ab,kw OR ‘toripalimab’:ti,ab,kw OR ‘dostarlimab’:ti,ab,kw

- Concept 2: Ophthalmologic

‘Eye’/exp OR ‘eye*’:ti,ab,kw OR ‘ophthalm*’:ti,ab,kw OR ‘ocular’:ti,ab,kw OR ‘oculomotor’:ti,ab,kw OR ‘ocular adnexa’:ti,ab,kw OR ‘cornea*’:ti,ab,kw OR ‘uvea*’:ti,ab,kw OR ‘retina*’:ti,ab,kw OR ‘conjunctiva*’:ti,ab,kw OR ‘iris’:ti,ab,kw OR ‘ciliary body’:ti,ab,kw OR ‘trabecular meshwork’:ti,ab,kw OR ‘choroid*’:ti,ab,kw OR ‘lens’:ti,ab,kw OR ‘optic’:ti,ab,kw OR ‘orbit*’:ti,ab,kw OR ‘vitreous’:ti,ab,kw OR ‘vitreum’:ti,ab,kw OR ‘sclera*’:ti,ab,kw OR ‘extraocular muscle*’:ti,ab,kw OR ‘intraocular pressure’:ti,ab,kw OR ‘lacrimal’:ti,ab,kw

- Concept 3: Adverse events

‘Adverse drug reaction’/exp OR ‘Adverse drug reaction’:ti,ab,kw OR ‘adverse effect*’:ti,ab,kw OR ‘side effect*’:ti,ab,kw OR ‘complication*’:ti,ab,kw OR ‘adverse reaction*’:ti,ab,kw OR ‘toxic*’:ti,ab,kw OR 'eye disease'/exp OR ‘orbitopath*’:ti,ab,kw OR ‘ophthalmopathy*’:ti,ab,kw OR ‘inflammat*’:ti,ab,kw OR ‘cellulitis’:ti,ab,kw OR ‘Graves’:ti,ab,kw OR ‘thyroid eye disease’:ti,ab,kw OR ‘tolosa hunt’:ti,ab,kw OR ‘myopath*’:ti,ab,kw OR ‘myositis’:ti,ab,kw OR ‘palsy’:ti,ab,kw OR ‘palsies’:ti,ab,kw OR ‘paralysis’:ti,ab,kw OR ‘myoplegia’:ti,ab,kw OR ‘ophthalmoplegia’:ti,ab,kw OR ‘myasthenia’:ti,ab,kw OR 'myasthenia gravis'/exp OR ‘uveitis’:ti,ab,kw OR ‘panuveitis’:ti,ab,kw OR ‘planitis’:ti,ab,kw OR ‘vitritis’:ti,ab,kw OR ‘iritis’:ti,ab,kw OR ‘keratouveitis’:ti,ab,kw OR ‘iridocyclitis’:ti,ab,kw OR ‘white dot syndrome*’:ti,ab,kw OR ‘episcleritis’:ti,ab,kw OR ‘scleritis’:ti,ab,kw OR ‘conjunctivitis’:ti,ab,kw OR ‘dry eye*’:ti,ab,kw OR ‘sicca’:ti,ab,kw OR ‘Sjogren’:ti,ab,kw OR ‘Sjoegren’:ti,ab,kw OR 'Sjoegren syndrome'/exp OR ‘retinitis’:ti,ab,kw OR ‘chorioretinitis’:ti,ab,kw OR ‘vitreoretinopath*’:ti,ab,kw OR ‘chorioretinopath*’:ti,ab,kw OR ‘retinopath*’:ti,ab,kw OR ‘maculopath*’:ti,ab,kw OR ‘drusen*’:ti,ab,kw OR ‘detachment’:ti,ab,kw OR ‘neovascularization’:ti,ab,kw OR ‘vasculopath*’:ti,ab,kw OR ‘epiretinal membrane’:ti,ab,kw OR ‘vasculitis’:ti,ab,kw OR ‘arteritis’:ti,ab,kw OR ‘arteriitis’:ti,ab,kw OR 'giant cell arteritis'/exp OR ‘necrosis’:ti,ab,kw OR ‘hemorrhag*’:ti,ab,kw OR ‘occlusion’:ti,ab,kw OR ‘neuroretinitis’:ti,ab,kw OR ‘neuroretinopath*’:ti,ab,kw OR ‘choroiditis’:ti,ab,kw OR ‘effusion*’:ti,ab,kw OR ‘choroideremia’:ti,ab,kw OR ‘choroidopath*’:ti,ab,kw OR ‘sarcoidosis’:ti,ab,kw OR 'sarcoidosis'/exp

OR ‘papillitis’:ti,ab,kw OR ‘papilledema’:ti,ab,kw OR ‘edema’:ti,ab,kw OR ‘glaucoma’:ti,ab,kw OR ‘neuropath*’:ti,ab,kw OR ‘neuritis’:ti,ab,kw OR ‘atrophy’:ti,ab,kw OR ‘neuromyelitis’:ti,ab,kw OR ‘nystagmus’:ti,ab,kw OR ‘strabismus’:ti,ab,kw OR ‘keratoconus’:ti,ab,kw OR ‘keratitis’:ti,ab,kw OR ‘ulcer*’:ti,ab,kw OR ‘infectio*’:ti,ab,kw OR ‘epitheliopath*’:ti,ab,kw OR ‘endophthalmitis’:ti,ab,kw OR ‘graft rejection’:ti,ab,kw OR ‘graft rejection’/exp OR ‘hypotension’:ti,ab,kw OR ‘hypertension’:ti,ab,kw OR ‘cataract’:ti,ab,kw OR ‘opacit*’:ti,ab,kw OR ‘dacryocystitis’:ti,ab,kw

Web of Science

- Concept 1: Immune checkpoint inhibitors

“Checkpoint inhib*” OR “checkpoint block*” OR ipilimumab OR nivolumab OR pembrolizumab OR cemiplimab OR atezolizumab OR avelumab OR durvalumab OR tremelimumab OR ticilimumab OR tremelimumab[tiab] OR ticilimumab[tiab] OR "tremelimumab"[supplementary Concept] OR spartalizumab[tiab] OR camrelizumab[tiab] OR sintilimab[tiab] OR tislelizumab[tiab] OR toripalimab[tiab] OR dostarlimab[tiab]

- Concept 2: Ophthalmologic

Eye* OR ophthalm* OR ocular OR oculomotor OR “ocular adnexa” OR cornea* OR uvea* OR retina* OR conjunctiva* OR iris OR “ciliary body” OR “trabecular meshwork” OR choroid* OR lens OR optic OR orbit* OR vitreous OR vitreum OR sclera* OR “extraocular muscle*” OR “intraocular pressure” OR lacrimal

- Concept 3: Adverse events

“Adverse effect*” OR “adverse reaction*” OR “side effect*” OR complication* OR toxic* OR orbitopath* OR ophthalmopath* OR inflammat* OR cellulitis OR Graves OR “thyroid eye disease” OR “tolosa hunt” OR myopath* OR myositis OR palsy OR palsies OR paralysis OR myoplegia OR ophthalmoplegia OR myasthenia OR uveitis OR panuveitis OR planitis OR vitritis OR iritis OR keratouveitis OR iridocyclitis OR “white dot syndrome*” OR episcleritis OR scleritis OR conjunctivitis OR “dry eye*” OR sicca OR Sjogren* OR Sjoegren* OR retinitis OR chorioretinitis OR vitreoretinopath* OR chorioretinopath* OR retinopath* OR maculopath* OR drusen* OR detachment OR neovascularization OR vasculopath* OR “epiretinal membrane*” OR vasculitis OR arteritis OR arteriitis OR necrosis OR hemorrhag* OR occlusion OR neuroretinitis OR neuroretinopath* OR choroiditis OR effusion* OR choroideremia OR choroidopath* OR sarcoidosis OR papillitis OR papilledema OR edema OR glaucoma OR neuropath* OR neuritis OR atrophy OR neuromyelitis OR nystagmus OR strabismus OR keratoconus OR keratitis OR ulcer* OR infectio* OR epitheliopath* OR endophthalmitis OR “graft rejection” OR hypotension OR hypertension OR cataract OR opacit* OR dacryocystitis
