# Supplementary material for: Ocular adverse events associated with immune checkpoint inhibitors, a scoping review
Source: J Ophthalmic Inflamm Infect. 2023 Feb 22;13:5. doi: 10.1186/s12348-022-00321-2 (PMC9947214; doi:10.1186/s12348-022-00321-2)
Supplement: Supplementary file 2 — Additional file 2. [file 12348_2022_321_MOESM2_ESM.pdf]

Overview of all included case reports

Summary of all case reports related to melanoma.

References	ICI used	Age	M/F	Ethnicity	Cancer	Ocular toxicity	Time to event	Antitumor efficacy of ICI	Other irAEs	Treatment	Resolution	ICI discontinued
**Cornea and ocular surface**												
**Baughman et al. (2017)**	Nivolumab	92	F	NR	Metastatic cutaneous melanoma	Bilateral anterior uveitis and keratitis	After third dose	NR	NR	Topical steroids, cyclopentolate, tobramycine/dexamethasone ointment, artificial tears	Y	N
**Kim et al. (2019)**	Nivolumab	45	F	NR	Metastatic cutaneous melanoma	Bilateral dry eyes	After 2 cycles	Complete response	Y	Topical cyclosporine	Y	N
**Kim et al. (2019)**	Ipilimumab and nivolumab; Nivolumab	58	M	NR	Metastatic cutaneous melanoma	Unilateral atraumatic corneal perforation	After 8 cycles	Progressive cancer	Y	Corneal glue, bandage contact lens, artificial tears, Topical cyclosporine	Y	Y
**Nguyen et al. (2016)**	Nivolumab	58	M	NR	Metastatic melanoma	Dry eyes; Perforated cornea	After 6 cycles	Progressive cancer	NR	Artificial tears; punctum plugs, cyclosporine; corneal glue, bandage contact lens	Partially	Y
**Nguyen et al. (2016)**	Nivolumab	46	F	NR	Metastatic cutaneous melanoma	Dry eyes	After 3 cycles	Good response	NR	Artificial tears; topical cyclosporine	Y	Y
**Papavasileiou et al. (2016)**	Ipilimumab	55	F	NR	Metastatic melanoma	Bilateral peripheral ulcerative keratitis	After 3 cycles	Progressive cancer	N	Acyclovir PO, erythromycin oitnment; Topical steroids	Y	Y
**Parker et al. (2019)**	Nivolumab	80	F	Caucasian	Metastatic melanoma	Unilateral corneal ulceration	4m after R/ initiation	NR	Y	Ganciclovir gel; loteprednol etabonate gel; conjunctival flap procedure; valcyclovir PO; Topical profloxacin; Topical prednisolone	Y	Y
**Shahzad et al. (2021)**	Nivolumab	81	M	NR	Metastatic melanoma	Conjunctivitis, Left RD; Anterior uveitis	18m after R/ initiation	Complete response	NR	Topical steroids, Topical cyclopentolate	N	N
**Thomas et al. (2019)**	Pembrolizumab	88	M	Caucasian	Metastatic cutaneous melanoma	Bilateral corneal erosions as part of a Behçet's-like syndrome	24m after R/ initation	NR	Y	Topical and IV steroids; colchicine	Y	Y

NR = not reported, M = male, F = female, Y = yes, N = no, NSCLC = non small cell lung carcinoma, RCC = renal cell carcinoma, IOP = intraocular pressure, RD = retinal detachment, CME = cystoid macular edema, ON = optic nerve, CNV = choroidal neovascularization, RAPD = relative afferent pupil defect, AMN = acute macular neuroretinopathy, ARMD = Age-related macular degeneration, R/ = treatment, PO = per os, IV = intravenous, IVT = intravitreal, IVIG = intravenous immuneglobulines, PLEX = plasma exchange, VTX = vitrectomy

**Voskens et al. (2012)**	Ipilimumab	53	F	NR	Metastatic mucosal melanoma	Sjögren syndrome; Iridocyclitis, marginal keratitis	2m after R/ initiation	Partial respons e	Y	Systemic steroids, local therapy	Y	Y
**Voskens et al. (2013)**	Ipilimumab	57	M	NR	Metastatic cutaneous melanoma	Conjunctivitis	12w after R/ initiation	Partial respons e	NR	Sodium hyaluronate eye gel	Y	NR
**Warner et al. (2019)**	Ipilimumab and pembrolizumab; Pembrolizumab	65	M	NR	Metastatic melanoma	Sicca Syndrome: mild to moderate sialadenitis	54d after R/ initiation	Complet e respons e	Y	PO steroids	Partially	N
**Warner et al. (2019)**	Nivolumab	71	M	NR	Metastatic melanoma	Sicca Syndrome: mild to moderate sialoadenitis	76d after R/ initiation	Non-CR/Non -PD	N	PO steroids	Partially	Y
**Warner et al. (2019)**	Pembrolizumab	59	M	NR	Metastatic melanoma	Sicca Syndrome: mild chronic sialadenitis	152d after R/ initiation	Complet e respons e	Y	None	N/A	Y
**Warner et al. (2019)**	Nivolumab	52	M	NR	Metastatic melanoma	Sicca Syndrome: mild chronic sialadenitis	63d after R/ initiation	Stable disease	Y	PO steroids	Partially	Y
**Uveitis**												
**Abu Samra et al. (2016)**	Pembrolizumab	82	M	NR	Metastatic cutaneous melanoma	Bilateral anterior uveitis and papillitis	2m after R/ initiation	NR	N	Topical and PO steroids	NR	Y
**Acaba-Berrocal et al. (2018)**	Pembrolizumab	65	F	Cauc asian	Metastatic cutaneous melanoma	Bilateral HLA-A29-negative Birdshot-like chorioretinopathy	2y after R/ initiation	NR	NR	Periocular triamcinolone injection	Partially	N
**Arai et al. (2016)**	Nivolumab	65	M	Asian	Metastatic cutaneous melanoma	Bilateral acute anterior uveitis and VKH-syndrome like eruptions	2w after R/ initiation	NR	Y	Topical steroids and mydriatics	Y	N
**Basilious et al. (2016)**	Pembrolizumab	63	F	NR	Metastatic cutaneous melanoma	Bilateral anterior uveitis resulting in severe hypotony and the development of PSC; after cataract Sx: CME and intraretinal and subretinal fluid as well as hypotony maculopathy	NR	NR	NR	Topical steroids and homatropine, PO steroids - cataract Sx - IVT steroids	Partially	Y
**Baughman et al. (2017)**	Nivolumab	92	F	NR	Metastatic cutaneous melanoma	Bilateral anterior uveitis and keratitis	After third dose	NR	NR	Topical steroids, cyclopentolate, tobramycine/dexamethasone ointment, artificial tears	Y	N
**Bitton et al. (2019)**	Pembrolizumab	44	F	NR	Malignant melanoma	Bilateral anterior uveitis	After 18 cycles	Stable disease	Y	Topical steroids	Y	Y

NR = not reported, M = male, F = female, Y = yes, N = no, NSCLC = non small cell lung carcinoma, RCC = renal cell carcinoma, IOP = intraocular pressure, RD = retinal detachment, CME = cystoid macular edema, ON = optic nerve, CNV = choroidal neovascularization, RAPD = relative afferent pupil defect, AMN = acute macular neuroretinopathy, ARMD = Age-related macular degeneration, R/ = treatment, PO = per os, IV = intravenous, IVT = intravitreal, IVIG = intravenous immuneglobulines, PLEX = plasma exchange, VTX = vitrectomy

**Bitton et al. (2019)**	Pembrolizumab	71	F	NR	Malignant melanoma	Bilateral anterior uveitis	After 8 cycles	Partial respons e	Y	Topical steroids	Y	N
**Bobek et al. (2013)**	Ipilimumab	81	M	NR	Metastatic cutaneous melanoma	Bilateral CNV in patient with ARMD	1y after R/ initation	Good respons e	Y	Intravitreal anti-VEGF	Partially (	Y
**Bricout et al. (2017)**	Pembrolizumab	59	M	Cauc asian	Metastatic cutaneous melanoma	Bilateral panuveitis with bilateral exudative RD with 360° choroidal detachments (VKH-like syndrome)	16m after R/ initiation	Partial respons e	Y	Topical, PO, IV steroids; subconjunctival steroids	Y (recurrence when tapering steroids)	Y
**Chan et al. (2017)**	Ipilimumab and nivolumab	74	M	NR	Metastatic choroidal melanoma	Bilateral anterior uveitis	3m after R/ initiation	NR	Y	Topical and PO steroids; anticholinergic eye drops	Y	Y
**Chang et al. (2018)**	Ipilimumab and pembrolizumab	65	M	NR	Metastatic melanoma	Bilateral anterior uveitis	After 3 cycles	Stable disease	Y	Topical steroids and atropine, PO steroids	Y	Y
**Conrady et al. (2018)**	Ipilimumab and nivolumab	71	F	NR	Metastatic cutaneous melanoma	Bilateral choroidal folds, ON hyperaemia, and SRF (VKH-like syndrome)	12w after R/ initiation	NR	Y	IV and PO Steroids	Y	NR
**Conrady et al. (2018)**	Pembrolizumab	78	F	Cauc asian	Metastatic cutaneous melanoma	Ciliary body detachment, choroidal effusions, panuveïtis; Retinal detachment	2w after R/ initiation	NR	NR	Sub-Tenon's triamcinolone injecties; RD repair with fluocinolone acetonide placement	Partially	NR
**Conrady et al. (2018)**	Pembrolizumab	57	M	NR	Metastatic cutaneous melanoma	Bilateral choroidal folds, ON hyperaemia, and SRF (VKH-like syndrome)	After 15 R/ cycles	NR	NR	PO steroids	Y	Y
**Crews et al. (2015)**	Ipilimumab	46	M	NR	Metastatic cutaneous melanoma	VKH-like syndrome: bilateral serous retinal detachments, fine nongranulomatous keratic precipitates	6w after R/ initiation	NR	NR	IV Steroids	Partially	Y
**Crosson et al. (2015)**	Ipilimumab	54	F	NR	Metastatic cutaneous melanoma	Bilateral diffuse choroidal hypopigmentation, signs of prior exudative RD (VKH-like syndrome) - inactive disease	1y following R/	Partial respons e	Y	None	Partially	N (finis hed)
**Cuadrad o et al. (2019)**	Nivolumab	75	F	NR	Metastatic cutaneous melanoma	Mild bilateral anterior uveitis	4m after R/ initiation	NR	NR	Topical steroids; IV followed by PO steroids	Y	N
**De Vries et al. (2020)**	Pembrolizumab	25	F	Cauc asian	Metastatic cutaneous melanoma	Bilateral panuveitis and serous RD	After 5 R/ cycles	NR	NR	Topical and PO steroids	NR	Y
**Deitch-Harel et al. (2020)**	Ipilimumab	58	M	NR	Metastatic melanoma	Unilateral anterior uveitis; bilateral anterior and intermediate uveitis	6w after R/ initiation	NR	NR	Topical and Systemic steroids	Y	N

NR = not reported, M = male, F = female, Y = yes, N = no, NSCLC = non small cell lung carcinoma, RCC = renal cell carcinoma, IOP = intraocular pressure, RD = retinal detachment, CME = cystoid macular edema, ON = optic nerve, CNV = choroidal neovascularization, RAPD = relative afferent pupil defect, AMN = acute macular neuroretinopathy, ARMD = Age-related macular degeneration, R/ = treatment, PO = per os, IV = intravenous, IVT = intravitreal, IVIG = intravenous immuneglobulines, PLEX = plasma exchange, VTX = vitrectomy

**Diem et al. (2016)**	Pembrolizumab	60	NR	NR	Metastatic cutaneous melanoma	Bilateral panuveitis after elective unilateral cataract Sx	>3m after R/ initiation	NR	NR	Topical and PO steroids; topical antibiotics	Y	Y
**Dimitriou et al. (2020)**	Ipilimumab	33	M	NR	Advanced cutaneous melanoma	Uveitis (Anterior + intermediate)	52d after R/ initiation	Partial response	Y	NR	NR	NR
**Dimitriou et al. (2020)**	Ipilimumab AND pembrolizumab or nivolumab	55	M	NR	Advanced cutaneous melanoma	Uveitis (Anterior + intermediate + retinal)	154d after R/ initiation	Stable disease	Y	NR	NR	NR
**Dimitriou et al. (2020)**	Ipilimumab AND pembrolizumab or nivolumab	73	F	NR	Advanced cutaneous melanoma	Bilateral anterior uveitis	62d after R/ initiation	Partial response	N	NR	NR	NR
**Dimitriou et al. (2020)**	Ipilimumab	43	M	NR	Advanced cutaneous melanoma	Anterior + intermediate + optic nerve uveitis	45d after R/ initiation	Partial response	Y	NR	NR	NR
**Dolaghan et al. (2019)**	Pembrolizumab	72	M	NR	Metastatic cutaneous melanoma	Bilateral anterior uveitis with ocular hypertension; Melanoma-associated Retinopathy (MAR)	After 5 cycles	Complete response	Y	Topical and PO steroids	N	Y
**Dolaghan et al. (2019)**	Pembrolizumab	77	F	NR	Metastatic cutaneous melanoma	Bilateral panuveitis with exudative RD (VKH-like syndrome)	After 2 R/ cycles	NR	NR	Topical and PO steroids	NR	N
**Enomoto et al. (2021)**	Pembrolizumab	68	F	NR	Metastatic melanoma	VKH-like uveitis: ciliary body edema, SRD	5m after R/ initiation	Complete response	Y	Topical and PO steroids	Y	Y
**Fierz et al. (2016)**	Ipilimumab	43	M	NR	Metastatic cutaneous melanoma	Bilateral panuveitis with ocular hypertension	3w after second dose	NR	NR	Topical and PO steroids; IOP lowering drugs	Y	Y
**Fierz et al. (2016)**	Nivolumab	54	M	NR	Metastatic cutaneous melanoma	Bilateral panuveitis	1m after R/ initiation with vemurafenib	NR	NR	Prednisolone acetate and scopolamine	Y	Y
**Francis et al. (2020)**	Ipilimumab and nivolumab	61	F	NR	Cutaneous melanoma	Bilateral optic neuritis; Bilateral anterior uveitis	After 3 cycles	NR	NR	Topical and PO steroids; timolol plus dorzolamide	N	Y
**Francis et al. (2020)**	Nivolumab	63	M	NR	Cutaneous melanoma	Bilateral optic neuritis; Bilateral anterior uveitis	After 10 cycles	NR	NR	IV and PO steroids; topical difluprednate, timolol, plus dorzolamide, brimonidine	N	Y
**Fujimura et al. (2018)**	Nivolumab	73	M	Asian	Metastatic cutaneous melanoma	VKH-like syndrome: Bilateral serous RD with bilateral diffuse thickening of choroid	3m after R/ initiation with BRAF-inhibitors	Complete response	Y	IV followed by PO steroids	Y	Y

NR = not reported, M = male, F = female, Y = yes, N = no, NSCLC = non small cell lung carcinoma, RCC = renal cell carcinoma, IOP = intraocular pressure, RD = retinal detachment, CME = cystoid macular edema, ON = optic nerve, CNV = choroidal neovascularization, RAPD = relative afferent pupil defect, AMN = acute macular neuroretinopathy, ARMD = Age-related macular degeneration, R/ = treatment, PO = per os, IV = intravenous, IVT = intravitreal, IVIG = intravenous immuneglobulines, PLEX = plasma exchange, VTX = vitrectomy

**Fujimura et al. (2018)**	Nivolumab	35	F	Asian	Metastatic cutaneous melanoma	VKH-like syndrome: Bilateral serous RD	3m after R/ Re-administration nivolumab; 5m after R/ BRAF- inhibitors	Complet e respons e	Y	IV Steroids	Y	Y
**Gambichler et al. (2020)**	Nivolumab	63	F	NR	Metastatic cutaneous melanoma	VKH-like syndrome: bilateral disc edema, uveitis anterior/intermedia	After 3 cycles	Complet e respons e	Y	IV and PO Steroids	Y	Y
**Hahn et al. (2016)**	Ipilimumab	44	M	Hispa nic	Metastatic cutaneous melanoma	Bilateral anterior uveitis and neuroretinitis	2m after third dose	NR	Y	Topical and PO steroids	Partially	Y
**Hanna et al. (2016)**	Pembrolizumab	78	F	Cauc asian	Metastatic cutaneous melanoma	Bilateral panuveitis with serous choroidal effusions and CB failure	12d after R/ initiation - progressive worsening	NR	NR	PO and STN steroids	Y	Y
**Kanno et al. (2017)**	Nivolumab	54	F	NR	Metastatic melanoma	Bilateral anterior uveitis	After 4 cycles	Tumor respons e	Y	Topical steroids	Y	N
**Kim et al. (2019)**	Ipilimumab and nivolumab	71	F	NR	Metastatic uveal melanoma	Bilateral anterior uveitis	After 2 cycles	Progres sive cancer	Y	Topical and Systemic steroids	Y	N
**Kim et al. (2019)**	Ipilimumab and nivolumab	74	M	NR	Metastatic cutaneous melanoma	Bilateral anterior uveitis	After 2 cycles	Partial respons e	Y	Topical and Systemic steroids; topical atropine	Partially	N
**Kim et al. (2019)**	Nivolumab; Ipilimumab and nivolumab	53	F	NR	Metastatic cutaneous melanoma	Bilateral anterior uveitis and vitritis	After 10 cycles	Progres sive cancer	Y	Topical and Systemic steroids	N	Y
**Kim et al. (2019)**	Ipilimumab and nivolumab	67	M	NR	Metastatic cutaneous melanoma	Bilateral anterior uveitis	After 3 cycles	Progres sive cancer	Y	IVT, topical and Systemic steroids	N	Y
**Kim et al. (2019)**	Ipilimumab and nivolumab	69	M	NR	Metastatic cutaneous melanoma	Bilateral anterior uveitis and choroidal effusion	After 4 cycles	Partial respons e	Y	Topical and Systemic steroids	N	Y
**Kim et al. (2019)**	Nivolumab; Ipilimumab	68	F	NR	Metastatic cutaneous melanoma	Sarcoidosis-like uveitis, Bilateral cystoid macular edema	After 7 cycles	Stable disease	Y	Systemic steroids	NR	Y
**Kim et al. (2019)**	Ipilimumab and nivolumab	43	F	NR	Metastatic cutaneous melanoma	VKH-like syndrome	After 1 cycle	Progres sive cancer	Y	Systemic steroids	Y	Y
**Kiratli et al. (2016)**	Ipilimumab	43	F	NR	Metastatic choroidal melanoma	Bilateral anterior uveitis	After 3 cycles	NR	NR	Topical steroids	Y	NR

NR = not reported, M = male, F = female, Y = yes, N = no, NSCLC = non small cell lung carcinoma, RCC = renal cell carcinoma, IOP = intraocular pressure, RD = retinal detachment, CME = cystoid macular edema, ON = optic nerve, CNV = choroidal neovascularization, RAPD = relative afferent pupil defect, AMN = acute macular neuroretinopathy, ARMD = Age-related macular degeneration, R/ = treatment, PO = per os, IV = intravenous, IVT = intravitreal, IVIG = intravenous immuneglobulines, PLEX = plasma exchange, VTX = vitrectomy

**Kwek et al. (2016)**	Ipilimumab	NR	NR	NR	Metastatic cutaneous melanoma	Unilateral anterior uveitis; unilateral retinitis and ON swelling	9m after R/ initation	NR	NR	Topical steroids	Partially	Y
**Lau et al. (2019)**	Ipilimumab and nivolumab	68	M	NR	Metastatic cutaneous melanoma	Unilateral Listeria endophthalmitis, initially misdiagnosed as panuveitis (R/ with IVT steroids)	1m after stop nivolumab	NR	NR	VTX; IVT and systemic AB	NR	NR
**Liao et al. (2014)**	Ipilimumab	62	M	NR	Metastatic uveal melanoma	Uveitis	After 2 cycles	Progressive cancer	Y	IV Steroids	NR	Y
**Lise et al. (2016)**	Pembrolizumab	68	F	NR	Metastatic melanoma	Panuveitis with multifocal choroiditis; Systemic sarcoidosis	6m after R/ initiation	NR	NR	NR	NR	NR
**Manusow et al. (2014)**	Pembrolizumab	36	F	NR	Metastatic cutaneous melanoma	Unilateral retinal vasculitis and ocular vitreous metastasis	After 3 cycles (9w); after 15 cycles (+/- 10m)	Complete response	Y	Topical steroids; diagnostic VTX	Y	N (finished)
**Matsuo et al. (2015)**	Nivolumab	60	F	Asian	Metastatic cutaneous melanoma	Bilateral choroiditis (VKH-like syndrome)	3w after second dose (6w after R/ initiation)	Partial response	Y	Topical and PO steroids	Y	N
**McDonald et al. (2018)**	Ipilimumab and nivolumab	58	F	Caucasian	Metastatic cutaneous melanoma	VKH-like syndrome: uveitis, exudative retinal detachments	After 4 cycles	Progressive cancer	NR	Topical and PO steroids	N	N (finished)
**Mihailovic et al. (2019)**	Nivolumab and ipilimumab	68	F	NR	Metastatic cutaneous melanoma	Bilateral panuveitis (VKH-like syndrome)	NR	NR	NR	Topical and PO steroids	NR	NR
**Miserocchi et al. (2015)**	Ipilimumab	71	F	Caucasian	Metastatic uveal melanoma	Bilateral anterior uveitis	2w after last (4th) dose	NR	Y	Topical steroids and tropicamide	Y	N (finished)
**Modjtahedi et al. (2013)**	Ipilimumab	81	M	NR	Metastatic melanoma	Bilateral choroidal neovascularization membrane: subretinal hemorrhagen, macular drusen	1y after R/ initation	Stable disease	Y	IVT ranibizumab or bevacizumab	Partially	Y
**Montaudié et al. (2016)**	Nivolumab	56	M	NR	Metastatic melanoma	Sarcoid-like granulomatosis	After 2 cycles	Progressive cancer	NR	PO steroids	Y	N
**Nallapaneni et al. (2014)**	Ipilimumab	62	M	NR	Metastatic cutaneous melanoma	Bilateral anterior uveitis	After 4 cycles	Partial response	Y	Topical and PO steroids	Y	Y
**Navarro-Perea et al. (2019)**	Pembrolizumab	38	M	Caucasian	Metastatic cutaneous melanoma	Bilateral uveitis and papillitis	After 6 cycles	NR	NR	Topical and PO steroids; cyclopentolate, tropicamide and phenylephrine eye drops	Y	Y

NR = not reported, M = male, F = female, Y = yes, N = no, NSCLC = non small cell lung carcinoma, RCC = renal cell carcinoma, IOP = intraocular pressure, RD = retinal detachment, CME = cystoid macular edema, ON = optic nerve, CNV = choroidal neovascularization, RAPD = relative afferent pupil defect, AMN = acute macular neuroretinopathy, ARMD = Age-related macular degeneration, R/ = treatment, PO = per os, IV = intravenous, IVT = intravitreal, IVIG = intravenous immuneglobulines, PLEX = plasma exchange, VTX = vitrectomy

**Noble et al. (2020)**	Pembrolizumab	40	M	Asian	Metastatic melanoma	Intermediate uveitis: trace vetreous cell with no evidence of vaculitis or CME	24w after R/ initiation	Progressive cancer	NR	Topical steroids	NR	Y
**Noble et al. (2020)**	Ipilimumab and nivolumab	56	M	Caucasian	Metastatic melanoma	Anterior uveitis (non-granulomatous) OS; Panuveitis OD: cystoid macular edema	20w after R/ initiation	NR	NR	Topical and local steroids	NR	Y
**Noble et al. (2020)**	Ipilimumab and nivolumab	62	F	Caucasian	Metastatic choroidal melanoma	VKH-like syndrome	4w after R/ initiation	NR	Y	PO steroids	Y	N
**Noble et al. (2020)**	Pembrolizumab	63	M	Caucasian	Metastatic melanoma	VKH-like syndrome	8w after R/ initiation	NR	NR	Topical steroids	Y	N
**Noble et al. (2020)**	Ipilimumab	30	F	Caucasian	Metastatic melanoma	VKH-like syndrome: recurrent bilateral anterior uveitis	18w after R/ initiation	NR	Y	Topical and PO steroids	Y	Y
**O'Bryhim et al. (2018)**	Ipilimumab and pembrolizumab	77	M	Caucasian	Metastatic cutaneous melanoma	Bilateral choroidal detachments	2w after R/ initiation	NR	N/R	Topical and PO steroids	Partially	Y
**Obata et al. (2019)**	Nivolumab	63	F	Asian	Metastatic cutaneous melanoma	Bilateral panuveitis (VKH-like syndrome)	10d after second dose (1m after R/ initiation)	NR	Y	Topical steroids and tropicamide	Y	N
**Papavasileiou et al. (2016)**	Ipilimumab	61	F	NR	Metastatic melanoma	Bilateral anterior uveitis	After 2 cycles	Progressive cancer	Y	Topical steroids	Y	Y
**Papavasileiou et al. (2016)**	Ipilimumab	54	F	NR	Metastatic melanoma	Bilateral anterior uveitis	After 2 cycles	Progressive cancer	Y	Topical steroids	Y	Y
**Patel et al. (2018)**	anti-PD-L1 (MPDL3280A)	71	M	Caucasian	Metastatic cutaneous melanoma	Panuveitis with bilateral CME	1y after R/ initation	Progressive cancer	NR	Topical and PO steroids; Topical NSAID; IVT steroids	Y	Y
**Rapisuwon et al. (2019)**	Nivolumab and ipilimumab	60	F	NR	Metastatic choroidal melanoma	Unilateral central serous retinopathy with retinal detachment (VKH-like syndrome)	After 2 cycles	NR	Y	PO steroids, followed by infliximab and vedolizumab	Y	Y
**Reid et al. (2019)**	Pembrolizumab	73	M	Caucasian	Metastatic cutaneous melanoma	Bilateral panuveitis with serous choroidal effusions and CB failure followed by chronic hypotony	NR	NR	Y	PO steroids and acetazolamide; ocular VED injections in AC combined with peribulbar steroids; phaco-VTX with SO tamponade in OS	Partially	Y
**Richardson et al. (2017)**	Nivolumab	74	F	NR	Metastatic cutaneous melanoma	Bilateral uveitis: 2+ anterior chamber cell, CME, chorioretinal folds	5m after R/ initiation	NR	NR	Topical and PO steroids, topical cyclosporine; IVT steroids	Partially	Y
**Rodriguez et al. (2019)**	Ipilimumab	54	F	Caucasian	Stage III cutaneous melanoma	Multiple bilateral serous retinal detachments and mydriasis	1d after second infusion	NR	Y	PO steroids	Partially	Y

NR = not reported, M = male, F = female, Y = yes, N = no, NSCLC = non small cell lung carcinoma, RCC = renal cell carcinoma, IOP = intraocular pressure, RD = retinal detachment, CME = cystoid macular edema, ON = optic nerve, CNV = choroidal neovascularization, RAPD = relative afferent pupil defect, AMN = acute macular neuroretinopathy, ARMD = Age-related macular degeneration, R/ = treatment, PO = per os, IV = intravenous, IVT = intravitreal, IVIG = intravenous immuneglobulines, PLEX = plasma exchange, VTX = vitrectomy

**Shahzad et al. (2021)**	Ipilimumab and nivolumab	50	M	NR	Metastatic melanoma	Bilateral anterior uveitis	3w after R/ initiation	Partial response	Y	Topical steroids, Topical cyclopentolate	Y	Y
**Shahzad et al. (2021)**	Pembrolizumab	74	F	NR	Resected stage III melanoma	Intermediate uveitis	1w after R/ initiation	Progressive cancer	Y	PO steroids	Y	Y
**Sun et al. (2020)**	Ipilimumab	56	F	Caucasian	Metastatic cutaneous melanoma	Bilateral anterior uveitis, papillitis and CME	52d after R/ initiation	NR	N/R	Transseptal followed by retrobulbar steroid injections	Y	N
**Sun et al. (2020)**	Ipilimumab	43	F	Caucasian	Metastatic cutaneous melanoma	Bilateral posterior uveitis	63d after R/ initiation	NR	N/R	PO steroids	Partially	Y
**Sun et al. (2020)**	Ipilimumab	61	F	N/R	Metastatic cutaneous melanoma	Bilateral panuveitis	41d after R/ initiation	NR	N/R	Topical steroids	Y	N
**Sun et al. (2020)**	Ipilimumab	66	M	Caucasian	Metastatic cutaneous melanoma	Bilateral panuveitis	85d after R/ initiation	NR	N/R	Topical and PO steroids	Y	Y
**Sun et al. (2020)**	Ipilimumab and nivolumab	47	M	Caucasian	Metastatic cutaneous melanoma	Anterior uveitis (laterality NR)	114d after R/ initiation	NR	N/R	Topical steroids	Y	N
**Sun et al. (2020)**	Ipilimumab and nivolumab	71	M	Caucasian	Metastatic cutaneous melanoma	Bilateral anterior uveitis	63d after R/ initiation	NR	N/R	Topical steroids	Y	N
**Sun et al. (2020)**	Ipilimumab and nivolumab	53	F	Caucasian	Metastatic cutaneous melanoma	Bilateral anterior uveitis	126d after R/ initiation	NR	N/R	Topical steroids	Y	N
**Sun et al. (2020)**	Ipilimumab and nivolumab	52	M	Caucasian	Metastatic cutaneous melanoma	Bilateral panuveitis	4d after R/ initiation	NR	N/R	Topical steroids and cyclopentolate	Y	N
**Sun et al. (2020)**	Ipilimumab and nivolumab	60	M	Caucasian	Metastatic cutaneous melanoma	Bilateral anterior uveitis	161d after R/ initiation	NR	N/R	Topical steroids and atropine; already on PO steroids for hypophysitis	Y	N
**Sun et al. (2020)**	Nivolumab	47	M	Caucasian	Metastatic cutaneous melanoma	Bilateral panuveitis	N/R	NR	N/R	Topical and PO steroids	Y	N
**Sun et al. (2020)**	Pembrolizumab	45	M	Caucasian	Metastatic cutaneous melanoma	Panuveitis (laterality NR)	N/R	NR	N/R	Topical steroids	Y; continued elevated IOP due to NVG	N

NR = not reported, M = male, F = female, Y = yes, N = no, NSCLC = non small cell lung carcinoma, RCC = renal cell carcinoma, IOP = intraocular pressure, RD = retinal detachment, CME = cystoid macular edema, ON = optic nerve, CNV = choroidal neovascularization, RAPD = relative afferent pupil defect, AMN = acute macular neuroretinopathy, ARMD = Age-related macular degeneration, R/ = treatment, PO = per os, IV = intravenous, IVT = intravitreal, IVIG = intravenous immuneglobulines, PLEX = plasma exchange, VTX = vitrectomy

**Sun et al. (2020)**	Pembrolizumab	68	F	Caucasian	Metastatic cutaneous melanoma	Bilateral panuveitis	43d after R/ initiation	NR	N/R	Topical and STN steroids; VTX SO OS	N: persistent hypotony	Y
**Tan et al. (2018)**	Ipilimumab	27	F	NR	Metastatic choroidal melanoma	Bilateral posterior uveitis	After 3 cycles	Progressive cancer	Y	IV and PO steroids	Y	Y
**Taylor et al. (2016)**	Pembrolizumab	61	F	Caucasian	Metastatic melanoma	Bilateral panuveitis	3d after R/ initiation	Progressive cancer	Y	Topical and PO steroids	Partially	N
**Telfah et al. (2019)**	Pembrolizumab	58	M	NR	Metastatic cutaneous melanoma	Bilateral shallow choroidal effusions with exudative RD	After 14 cycles	Complete response	NR	Topical and systemic steroids	Y	Y
**Telfah et al. (2019)**	Pembrolizumab	57	M	NR	Metastatic cutaneous melanoma	Bilateral posterior uveitis with vitreous cell, choroidal folds, and right ON edema	6m after R/ initiation	NR	NR	Topical and systemic steroids	Y	Y
**Theillac et al. (2017)**	Nivolumab	55	M	Caucasian	Metastatic cutaneous melanoma	Bilateral anterior uveitis, papillitis and unilateral serous retinal detachment	After third dose	NR	NR	Topical and PO steroids	Y	Y
**Thomas et al. (2018)**	Nivolumab	52	M	Caucasian	Metastatic cutaneous melanoma	Bilateral anterior uveitis, serous choroidal detachment with dot-blot hemorrhages in the peripheral retina	After 2 cycles	NR	NR	Peripheral iridotomies; Topical steroids, brimonidine, dorzolamide, timolol, atropine	Y	Y
**Thomas et al. (2018)**	Pembrolizumab	85	M	Caucasian	Desmoplastic cutaneous melanoma	Macular striae, serous and bullous choroidal detachment	After 3 cycles	Progressive cancer	NR	NR	N	N
**Tsui et al. (2017)**	Ipilimumab and nivolumab	68	M	African-American	Metastatic cutaneous melanoma	Bilateral choroidal effusions and exsudative retinal detachment	5w after R/ initiation	NR	NR	PO steroids	Partially	Y
**Ung et al. (2020)**	Nivolumab	55	M	NR	Metastatic cutaneous melanoma	Bilateral sarcoid choroidal granuloma's	After 6 cycles	NR	Y	None	N	Y
**Venkat et al. (2020)**	Nivolumab and ipilimumab	50	F	NR	Metastatic cutaneous melanoma	Bilateral panuveitis	4d after R/ reinitiation	NR	Y	Topical steroids and cyclopentolate	Y	N
**Witmer et al. (2017)**	Ipilimumab	54	M	NR	Metastatic cutaneous melanoma	Bilateral panuveitis (VKH-like syndrome)	After 4 cycles	Partial response	Y	PO steroids	Y	N
**Wong et al. (2012)**	Ipilimumab	43	F	NR	Metastatic cutaneous melanoma	Bilateral vitritis, choroiditis and serous RD suggestive of VKH syndrome; poliosis	2w after R/ initiation	NR	NR	IV followed by PO steroids	Y	Y

NR = not reported, M = male, F = female, Y = yes, N = no, NSCLC = non small cell lung carcinoma, RCC = renal cell carcinoma, IOP = intraocular pressure, RD = retinal detachment, CME = cystoid macular edema, ON = optic nerve, CNV = choroidal neovascularization, RAPD = relative afferent pupil defect, AMN = acute macular neuroretinopathy, ARMD = Age-related macular degeneration, R/ = treatment, PO = per os, IV = intravenous, IVT = intravitreal, IVIG = intravenous immuneglobulines, PLEX = plasma exchange, VTX = vitrectomy

**Retina**												
**Audemard et al. (2013)**	Ipilimumab	70	F	NR	Metastatic cutaneous melanoma	Melanoma-associated Retinopathy		Stable disease	NR	Topical Steroids	NR	NR
**Bobek et al. (2013)**	Ipilimumab	81	M	NR	Metastatic cutaneous melanoma	Bilateral CNV in patient with ARMD	1y after R/ initation	Good response	Y	Intravitreal anti-VEGF	Partially	Y
**Canestraro et al. (2020)**	Ipilimumab and nivolumab; Pembrolizumab; Nivolumab	69	F	Caucasian	Metastatic cutaneous melanoma	Choroidal thinning and fundus depigmentation	during 26 months of FU	NR	Y	NR	NR	NR
**Dolaghan et al. (2019)**	Pembrolizumab	72	M	NR	Metastatic cutaneous melanoma	Bilateral anterior uveitis with ocular hypertension; Melanoma-associated Retinopathy (MAR)	After 5 cycles	Complete response	Y	Topical and PO steroids	N	Y
**Elwood et al. (2019)**	Ipilimumab and nivolumab	65	F	NR	Metastatic cutaneous melanoma	Bilateral chorioretinal atrophy, retinovascular leakage, and CNV (unilateral) < MAR	Since R/ initiation (presentation 14m after)	Complete response	Y	Bevacizumab OD, STN steroids OS	Partially	Y
**Kemels et al. (2020)**	Nivolumab	74	M	NR	Metastatic mucosal melanoma	AEPVM	3w after R/ initiation	Progressive cancer	NR	PO steroids	N	Y
**Kemels et al. (2020)**	Nivolumab	51	F	NR	Metastatic mucosal melanoma	AEPVM	1m after R/ initiation	Good response	Y	Local and PO steroids	N	Y
**Kim et al. (2019)**	Ipilimumab and nivolumab	79	F	NR	Metastatic cutaneous melanoma	Melanoma-associated Retinopathy	After 1 cycle	Complete response	Y	IV steroids, IVIG	Partially	Y
**Krohn et al. (2020)**	Nivolumab	61	M	Caucasian	Metastatic cutaneous melanoma	Bilateral fundus hypopigmentation and Loss of choroidal naevi pigmentation	2y after R/ initiation	Complete response	Y	None	/	N
**Lambert et al. (2021)**	Pembrolizumab	54	F	NR	Metastatic mucosal melanoma	AEPVM	After 4 cycles	Partial response	Y	None	Y	Y
**Mantopoulos et al. (2015)**	Ipilimumab	72	F	NR	Acral lentiginous melanoma	AEPVM	NR	NR	Topical and PO steroids	Y	Y	N
**Miyakubo et al. (2019)**	Ipilimumab	78	M	Asian	Metastatic cutaneous melanoma	AEPVM	After 2 cycles (at d22)	NR	NR	None	N	N

NR = not reported, M = male, F = female, Y = yes, N = no, NSCLC = non small cell lung carcinoma, RCC = renal cell carcinoma, IOP = intraocular pressure, RD = retinal detachment, CME = cystoid macular edema, ON = optic nerve, CNV = choroidal neovascularization, RAPD = relative afferent pupil defect, AMN = acute macular neuroretinopathy, ARMD = Age-related macular degeneration, R/ = treatment, PO = per os, IV = intravenous, IVT = intravitreal, IVIG = intravenous immuneglobulines, PLEX = plasma exchange, VTX = vitrectomy

**Miyamoto et al. (2020)**	Nivolumab	73	M	Asian	Metastatic melanoma	AEPVM	2m after R/ initiation	Progressive cancer	NR	None	N	N
**Poujade et al. (2021)**	Pembrolizumab	68	F	Caucasian	Metastatic melanoma	Melanoma-associated Retinopathy	Before R/ initiation	Stable disease	NR	IVT steroids	Partially	N
**Roberts et al. (2016)**	Pembrolizumab	NR		NR	Metastatic cutaneous melanoma	Atypical melanoma-associated retinopathy with chorioretinal lesions	NR	NR	NR	None	N	N
**Sandhu et al. (2019)**	Pembrolizumab	55	F	NR	Metastatic cutaneous melanoma	Acute exudative polymorphous vitelliform maculopathy	5d-3w after R/ initiation	NR	NR	Topical CS and dorzolamide	Y	N
**Shahzad et al. (2021)**	Ipilimumab and nivolumab	56	M	NR	Metastatic melanoma	Melanoma-associated Retinopathy	3w after R/ initiation	Partial response	Y	Topical and PO steroids, Anti-VEGF injections	N	Y
**Sophie et al. (2019)**	Nivolumab	59	M	Caucasian	Melanoma	Fundus hypopigmentation, loss of chroidal nevi pigmentation	10m after R/ initiation	NR	Y	NR	NR	Y
**Neuro-ophthalmic**												
**Algaeed et al. (2018)**	Pembrolizumab	73	M	NR	Melanoma	Myasthenia Gravis	3w after R/ initiation	NR	NR	Pyridostigmine; IVIG; Steroids; Plasmapheresis	Y	NR
**Algaeed et al. (2018)**	Pembrolizumab	62	M	NR	Melanoma	Myasthenia Gravis	3w after R/ initiation	NR	NR	Pyridostigmine; IVIG; Steroids; Plasmapheresis	Y	NR
**Alnahhas et al. (2016)**	Pembrolizumab	84	M	Caucasian	Metastatic melanoma	Myasthenia Gravis	3m after R/ initiation	Good response	NR	Systemic steroids, pyridostigmine, IVIG	NR	NR
**Alnahhas et al. (2016)**	Pembrolizumab	62	M	Caucasian	Metastatic melanoma	Myasthenia Gravis	3m after R/ initiation	Good response	NR	Systemic steroids, pyridostigmine, IVIG	NR	NR
**Dimitriou et al. (2020)**	Ipilimumab	43	M	NR	Advanced cutaneous melanoma	Anterior + intermediate + optic nerve uveitis	45d after R/ initiation	Partial response	Y	NR	NR	NR
**Dimitriou et al. (2020)**	Ipilimumab	61	M	NR	Advanced cutaneous melanoma	Anterior + intermediate + optic nerve uveitis	45d after R/ initiation	Partial response	Y	NR	NR	NR
**Earl et al. (2018)**	Pembrolizumab	74	M	NR	Metastatic melanoma	Myasthenia gravis exacerbation	After 2 cycles	NR	NR	PO steroids, mycophenolate mofetil, pyridostigmine, IVIG, plasmapheresis	Partially	Y

NR = not reported, M = male, F = female, Y = yes, N = no, NSCLC = non small cell lung carcinoma, RCC = renal cell carcinoma, IOP = intraocular pressure, RD = retinal detachment, CME = cystoid macular edema, ON = optic nerve, CNV = choroidal neovascularization, RAPD = relative afferent pupil defect, AMN = acute macular neuroretinopathy, ARMD = Age-related macular degeneration, R/ = treatment, PO = per os, IV = intravenous, IVT = intravitreal, IVIG = intravenous immuneglobulines, PLEX = plasma exchange, VTX = vitrectomy

**Fazel et al. (2019)**	Ipilimumab and nivolumab	78	F	NR	Malignant melanoma	Myositis, Myasthenia gravis	5d after R/ initiation	NR	Y	IV steroids; IVIG; Plasmapheresis	N	Y
**Francis et al. (2020)**	Ipilimumab and nivolumab	61	F	NR	Cutaneous melanoma	Bilateral optic neuritis; Bilateral anterior uveitis	After 3 cycles	NR	NR	Topical and PO steroids; timolol plus dorzolamide	N	Y
**Francis et al. (2020)**	Ipilimumab and nivolumab; Nivolumab	58	F	NR	Cutaneous melanoma	Bilateral Optic Neuritis	After 52 cycles	NR	NR	PO and IV steroids; IVIG; rituximab, PLEX	Partially	Y
**Francis et al. (2020)**	Ipilimumab and nivolumab	68	M	NR	Cutaneous melanoma	Unilateral Optic Neuritis	After 4 cycles	NR	NR	IV and PO Steroids	Partially	Y
**Francis et al. (2020)**	Ipilimumab and nivolumab	65	M	NR	Cutaneous melanoma	Unilateral Optic Neuritis	After 4 cycles	NR	NR	None	NA	Y
**Francis et al. (2020)**	Nivolumab	63	M	NR	Cutaneous melanoma	Bilateral optic neuritis; Bilateral anterior uveitis	After 10 cycles	NR	NR	IV and PO steroids; topical difluprednate, timolol, plus dorzolamide, brimonidine	N	Y
**Jaben et al. (2020)**	Nivolumab and ipilimumab; Pembrolizumab	64	M	NR	Metastatic cutaneous melanoma	Isolated abducens nerve palsy	13m after R/ initiation	Stable disease	Y	PO steroids	Y	Y
**Johnson et al. (2015)**	Ipilimumab	69	F	NR	Metastatic cutaneous melanoma	Myasthenia Gravis	After 2 cycles	Good respons e	Y	Pyridostigmine; IV steroids, plasmapheresis	Partially	NR
**Kim et al. (2019)**	Ipilimumab and nivolumab; Nivolumab	61	F	NR	Metastatic cutaneous melanoma	Unilateral Optic Neuritis	After 5 cycles	Progres sive cancer	Y	IV Steroids, IVIG, IV Infliximab	Partially	Y
**Kim et al. (2019)**	Ipilimumab and nivolumab	62	F	NR	Metastatic cutaneous melanoma	Myasthenia Gravis	After 1 cycle	Progres sive cancer	Y	Systemic steroids, IVIG	Partially	Y
**Lau et al. (2016)**	Pembrolizumab	75	M	NR	Metastatic melanoma	Myasthenia Gravis	5w after R/ initiation	Stable disease	NR	IV steroids, IVIG	Y	Y
**Liao et al. (2014)**	Ipilimumab	70	F	NR	Metastatic uveal melanoma	Myositis, Myasthenia gravis	After 2 cycles	Stable disease	Y	IV steroids, plasmapheresis; pyridostigmine	Y	NR
**Liu et al.**	Pembrolizumab	73	M	NR	Acral lentiginous melanoma	Myasthenia Gravis	After 2 cycles	Stable disease	NR	IV steroids, IVIG	Y	Y
**Maeda et al. (2016)**	Nivolumab	79	M	NR	Metastatic melanoma	Acute exacerbation of Myasthenia Gravis	After 3 cycles	Good respons e	NR	PO steroids (maintenance therapy)	Y	N

NR = not reported, M = male, F = female, Y = yes, N = no, NSCLC = non small cell lung carcinoma, RCC = renal cell carcinoma, IOP = intraocular pressure, RD = retinal detachment, CME = cystoid macular edema, ON = optic nerve, CNV = choroidal neovascularization, RAPD = relative afferent pupil defect, AMN = acute macular neuroretinopathy, ARMD = Age-related macular degeneration, R/ = treatment, PO = per os, IV = intravenous, IVT = intravitreal, IVIG = intravenous immuneglobulines, PLEX = plasma exchange, VTX = vitrectomy

**Makarious et al. (2017)**	Pembrolizumab	85	F	NR	Metastatic melanoma	Myasthenia Gravis	After 2 cycles	NR	NR	IVIG, steroids, pyridostigmine	Y	Y
**March et al. (2018)**	Pembrolizumab	63	M	Caucasian	Metastatic melanoma	Myasthenia Gravis	After 1 cycle	NR	NR	Systemic steroids, pyridostigmine; IVIG, Plasmapheresis	N	Y
**Montes et al. (2018)**	Ipilimumab	74	M	NR	Metastatic melanoma	Myasthenia Gravis	After 3 cycles	NR	Y	Systemic steroids, pyridostigmine	Partially	Y
**Nowosielski et al. (2020)**	Nivolumab; Ipilimumab	47	M	NR	Metastatic cutaneous melanoma	Encephalomyelitis with Optic Neuritis	After 3 cycles of Ipilimumab, After 13 cycles of Nivolumab	Complete response	Y	PO steroids; IV steroids; rituximab; methotrexate	Y	Y
**Ozarcuk et al. (2019)**	Pembrolizumab; Nivolumab and ipilimumab	61	M	Caucasian	Metastatic melanoma	Myasthenia Gravis	After 2 cycles of Nivolumab and ipilimumab	NR	Y	Pyridostigmine, Steroids; IVIG	Partially	NR
**Parikh et al. (2020)**	Ipilimumab and nivolumab	60	F	Caucasian	Melanoma	Optic nerve edema	5m after R/ initiation	Progressive cancer	Y	None	Y	Y
**Shirai et al. (2016)**	Nivolumab	81	F	NR	Metastatic melanoma	Myasthenia Gravis	After 1 cycle	NR	Y	IV steroids	N	Y
**Sun et al. (2020)**	Pembrolizumab	43	M	Caucasian	Metastatic cutaneous melanoma	Optic neuritis	NR	NR	NR	NR	NR	NR
**Vogrig et al. (2020)**	pembrolizumab	26	M	NR	Melanoma	Optic Neuropathy: RAPD (right)	1m after R/ initiation	Complete response	Y	None	Y	Y
**Vogrig et al. (2020)**	pembrolizumab	58	F	NR	Melanoma	Bilateral Optic Neuropathy	6m after R/ initiation	NR	NR	IV Steroids	N	Y
**Vogrig et al. (2020)**	Ipilimumab and pembrolizumab	75	F	NR	Melanoma	Bilateral Optic Atrophy	few weeks after R/ initiation	Stable disease	Y	IV Steroids and Plasma exchange (PLEX)	N	Y
**Vogrig et al. (2020)**	Ipilimumab and pembrolizumab	63	M	NR	Melanoma	Left abducens cranial nerve palsy	1m after R/ initiation	Partial response	Y	PO Steroids	Y	Y
**Vogrig et al. (2020)**	Nivolumab	82	M	NR	Melanoma	Left abducens cranial nerve palsy	After 3 cycles	Complete response	Y	PO Steroids	Y	Y
**Orbita and ocular adnexa**												

NR = not reported, M = male, F = female, Y = yes, N = no, NSCLC = non small cell lung carcinoma, RCC = renal cell carcinoma, IOP = intraocular pressure, RD = retinal detachment, CME = cystoid macular edema, ON = optic nerve, CNV = choroidal neovascularization, RAPD = relative afferent pupil defect, AMN = acute macular neuroretinopathy, ARMD = Age-related macular degeneration, R/ = treatment, PO = per os, IV = intravenous, IVT = intravitreal, IVIG = intravenous immuneglobulines, PLEX = plasma exchange, VTX = vitrectomy

**Alnabulsi et al. (2018)**	Ipilimumab and nivolumab	67	M	Caucasian	Metastatic melanoma	Ocular Myositis	10d after R/ initiation	NR	Y	IV Steroids, IVIG, IV Infliximab	N	NR
**Borodic et al. (2011)**	Ipilimumab	51	F	NR	Metastatic melanoma	Graves Orbitopathy	After 2 cycles	NR		Cantholysis and Steroids	Y	NR
**Haddox et al. (2016)**	Pembrolizumab	78	M	NR	Metastatic melanoma	Myopathy	After 2 cycles	Good response	N	Systemic steroids; PLEX	N	Y
**Hassanzadeh et al. (2018)**	Ipilimumab	64	F	Caucasian	Cutaneous melanoma	Orbital Apex syndrome: asymmetric enlargment of the right ocular muscles, right optic nerve compression, RAPD OD	NR	NR	N	IV and PO Steroids	Partially	Y
**Henderson et al. (2015)**	Ipilimumab	55	M	NR	Metastatic melanoma	Orbital inflammatory syndrome: blepharitis, chemosis, enlargement of the extraocular muscles	After 2 cycles	Complete response	Y	PO steroids	Partially	Y
**Lecouflet et al. (2013)**	Ipilimumab	67	F	NR	Metastatic cutaneous melanoma	Myositis	After 4 cycles	Complete response	NR	PO steroids	Y	N (finished)
**McElnea et al. (2014)**	Ipilimumab	68	F	Caucasian	Metastatic melanoma	Thyroid-like ophtalmopathy	After 3 cycles	NR	NR	IV and PO Steroids	Y	Y
**Min et al. (2011)**	Ipilimumab	51	F	NR	Metastatic melanoma	Graves' ophtalmopathy	After 4 cycles	NR	NR	IV and PO Steroids	Y	Y
**Nardin et al. (2019)**	Pembrolizumab	68	M	NR	Metastatic cutaneous melanoma	Inflammatory orbitopathy	After 51 cycles	Complete response	Y	IV Steroids	Y	Y
**Papavasileiou et al. (2016)**	Ipilimumab	68	F	NR	Metastatic melanoma	Orbital inflammation	After 1 cycle	Progressive cancer	Y	Systemic steroids	Y	Y
**Papavasileiou et al. (2016)**	Ipilimumab	52	M	NR	Metastatic melanoma	Orbital inflammation	After 3 cycles	Progressive cancer	Y	IV and PO steroids	Y	Y
**Papavasileiou et al. (2016)**	Ipilimumab	47	F	NR	Metastatic melanoma	Orbital inflammation	After 2 cycles	Progressive cancer	N	Systemic steroids	Y	Y
**Papavasileiou et al. (2016)**	Ipilimumab	78	M	NR	Metastatic melanoma	Orbital inflammation	After 2 cycles	Stable disease	Y	PO steroids	Partially	Y

NR = not reported, M = male, F = female, Y = yes, N = no, NSCLC = non small cell lung carcinoma, RCC = renal cell carcinoma, IOP = intraocular pressure, RD = retinal detachment, CME = cystoid macular edema, ON = optic nerve, CNV = choroidal neovascularization, RAPD = relative afferent pupil defect, AMN = acute macular neuroretinopathy, ARMD = Age-related macular degeneration, R/ = treatment, PO = per os, IV = intravenous, IVT = intravitreal, IVIG = intravenous immuneglobulines, PLEX = plasma exchange, VTX = vitrectomy

**Rhea et al. (2018)**	Ipilimumab; Pembrolizumab	83	M	NR	Metastatic cutaneous melanoma	Graves' ophtalmopathy	After 1 cycle	Complete response	Y	PO steroids	Y	Y
**Sagiv et al. (2019)**	Tremelimumab	51	M	NR	Metastatic cutaneous melanoma	Graves disease with associated Thyroid-like ophtalmopathy	6m after R/ initiation	Progressive cancer	NR	IV and PO steroids	Y	NR
**Sheldon et al. (2017)**	Ipilimumab	40	F	Caucasian	Metastatic cutaneous melanoma	Inflammatory orbitopathy	After 3 cycles	Complete response	Y	IV and PO steroids	Y	Y
**Sohrab et al. (2013)**	Ipilimumab	43	M	Caucasian	Metastatic uveal melanoma	Orbitopathy	After 6 cycles	NR	NR	PO steroids	Y	Y
**Vallet et al. (2016)**	Pembrolizumab	86	F	NR	Metastatic cutaneous melanoma	Myositis	After 2 cycles	Stable disease	NR	IV steroids; PLEX	Y	Y

Summary of all case reports related to lung cancer.

References	ICI used	Age	M/F	Ethnicity	Cancer	Ocular toxicity	Time to event	Antitumor efficacy of ICI	Other irAEs	Treatment	Resolution	ICI discontinued
**Cornea and ocular surface**												
**Bitton et al. (2019)**	Pembrolizumab	70	M	NR	Lung adenocarcinoma	Bilateral severe dry eye	After 2 cycles	Partial response	N	Topical steroids	Partially	N
**Horisberger et al. (2018)**	Nivolumab	67	M	NR	Metastatic lung adenocarcinoma	Bilateral sterile conjunctivitis	After 13 cycles	Complete response	Y	Topical steroids	Y	Y
**Kim et al. (2019)**	Ipilimumab and nivolumab	51	M	NR	Metastatic NSCLC	Conjunctival papillary reaction, bilateral subconjunctival hemorrhage	After 1 cycle	Partial response	Y	Topical neomycin/polymyxinB/Dexamethasone	Y	Y
**Le Fournis et al. (2016)**	Nivolumab	58	F	NR	Squamous NSCLC	Corneal graft rejection: conjunctival inflammation, diffuse superifcial keratitis, corneal graft edema	After 9 cycles	Good response	NR	IV, PO and topical steroids	N	Y
**Ramaekers et al. (2021)**	Pembrolizumab	68	M	NR	Lung adenocarcinoma	Bilateral corneal perforation	18m after R/ initiation	Partial response	Y	Tissue glue, bandage contact lens, topical moxifloxacin; penetrating keratoplasty; IV and PO steroids; Autologous serum	N	Y
**Uveitis**												
**Ahmad et al. (2019)**	Durvalumab	61	F	NR	Metastatic NSCLC	Bilateral anterior uveitis (Hx of bilateral panuveitis)	2m after R/ initiation	NR	NR	Topical steroids	Y	N

NR = not reported, M = male, F = female, Y = yes, N = no, NSCLC = non small cell lung carcinoma, RCC = renal cell carcinoma, IOP = intraocular pressure, RD = retinal detachment, CME = cystoid macular edema, ON = optic nerve, CNV = choroidal neovascularization, RAPD = relative afferent pupil defect, AMN = acute macular neuroretinopathy, ARMD = Age-related macular degeneration, R/ = treatment, PO = per os, IV = intravenous, IVT = intravitreal, IVIG = intravenous immuneglobulines, PLEX = plasma exchange, VTX = vitrectomy

**Andrade et al. (2020)**	Durvalumab	64	M	Caucasian	NSCLC	Sarcoidosis-like Retinal vasculitis with macular edema	20m after R/ initiation	NR	N	IV and PO Steroids	Y	N
**Bitton et al. (2019)**	Nivolumab and ipilimumab	77	M	NR	Mesothelioma	Bilateral anterior uveitis	After 2 cycles	Partial response	Y	Topical steroids	Y	Y
**Bitton et al. (2019)**	Nivolumab	63	F	NR	NSCLC	Bilateral panuveitis	After 36 cycles	Partial response	N	Topical steroids and Subconjunctival Steroid injections	Partially	N
**Demarkarian et al. (2020)**	Nivolumab	53	M	NR	Metastatic NSCLC	Bilateral panuveitis: temporal mottling of the retinal epithelium, vitreous haze	19 days after R/initiation	NR	NR	Steroids	Partially	Y
**Hefler et al. (2020)**	Ipilimumab and nivolumab	69	F	Caucasian	Metastatic SCLC	Bilateral panuveitis	After 1 cycle	Good response	Y	IVT steroids; topical cyclosporine, timolol, brimonidine	Y	Y
**Karlin et al. (2018)**	Nivolumab	66	F	NR	Metastatic NSCLC	Bilateral anterior uveitis	5m after R/ initiation	NR	NR	Topical steroids and cyclopentolate	Y	N
**Kim et al. (2021)**	Pembrolizumab	71	M	NR	Metastatic lung squamous cell carcinoma	Bilateral panuveitis and retinal vasculitis	2w after R/ initiation	Good response	Y	PO steroids; IVT steroids	Y	Y
**Kurono et al. (2020)**	Pembrolizumab	71	M	Asian	NSCLC	VKH-like syndrome: Bilateral granulomatous anterior uveitis with disc edema and multifocal serous RD	After 6 cycles	Partial response	Y	IV and PO Steroids	Y	N
**Lee et al. (2019)**	Pembrolizumab	77	M	Asian	Metastatic small-cell lung cancer	Recurrent sympathetic ophtalmia (VKH)	After third dose	NR	NR	Systemic steroids	Partially	Y
**Mimura et al. (2021)**	Pembrolizumab	71	M	NR	Metastatic squamous cell lung carcinoma	Uveitis with retinal vasculitis	After 1 cycle	Partial response	Y	Topical and PO steroids	Y	N
**Parikh et al. (2020)**	Nivolumab	66	F	African-American	NSCLC	Bilateral granulomatous anterior and intermediate uveitis and vitreouscells	8w after R/ initiation	NR	Y	Topical steroids	Y	Y
**Parikh et al. (2020)**	Durvalumab	71	F	Caucasian	NSCLC	Bilateral anterior uveitis	13m after R/ initiation	NR	NR	Topical steroids	Y	N
**Tamura et al. (2018)**	Pembrolizumab	61	M	Asian	Metastatic NSCLC	VKH-like syndrome	After 3 cycles	NR	Y	Steroids	Y	Y
**Thomas et al. (2018)**	Atezolizumab	68	M	African-American	Metastatic lung adenocarcinoma	Unilateral anterior uveitis, bullous serous choroidal detachment with a shallow exudative retinal detachment	1m after R/ initiation (after 2 cycles)	NR	NR	None	Y	Y
**Retina**												
**Ramtohul et al. (2020)**	Atezolizumab	52	M	NR	Metastatic NSCLC	Bilateral AMN with retinal venulitis	15d after R/ initiation	Pt died 4m later of progressive cancer	N	NR	Partially	Y

NR = not reported, M = male, F = female, Y = yes, N = no, NSCLC = non small cell lung carcinoma, RCC = renal cell carcinoma, IOP = intraocular pressure, RD = retinal detachment, CME = cystoid macular edema, ON = optic nerve, CNV = choroidal neovascularization, RAPD = relative afferent pupil defect, AMN = acute macular neuroretinopathy, ARMD = Age-related macular degeneration, R/ = treatment, PO = per os, IV = intravenous, IVT = intravitreal, IVIG = intravenous immuneglobulines, PLEX = plasma exchange, VTX = vitrectomy

**Ramtohul et al. (2020)**	Atezolizumab	42	M	NR	Metastatic NSCLC	Bilateral AMN	15d after R/ initiation	Pt died 6m later of progressive cancer	N	NR	Partially	Y
**Reddy et al. (2020)**	Nivolumab	64	F	Caucasian	Metastatic NSCLC	Immune retinopathy with photoreceptor toxicity	5m after R/ initiation	NR	Y	PO steroids	N	Y
**Neuro-ophthalmic disorders**												
**Chen et al. (2017)**	Nivolumab	65	M	NR	Squamous lung cell carcinoma	Myositis, Myasthenia gravis	2m after R/ initiation	NR	NR	Systemic steroids, pyridostigmine	N	NR
**Chen et al. (2017)**	ipilimumab and nivolumab	57	M	NR	Metastatic squamous cell carcinoma	Myasthenia gravis, myositis	4w after R/ initiation	NR	Y	IV steroids, PO pyridostigmine	Partially	NR
**Dhenin et al. (2019)**	Pembrolizumab	79	F	NR	Lung adenocarcinoma	Myasthenia gravis	After 6 cycles	Complete response	Y	IV steroids, PO pyridostigmine, Plasmapheresis	Y	Y
**Francis et al. (2020)**	Nivolumab	59	F	NR	SCLC	Unilateral Optic Neuritis	After 2 cycles	NR	NR	PO steroids	NA	Y
**Francis et al. (2020)**	Pembrolizumab	71	M	NR	NSCLC	Bilateral Optic Neuritis	After 3 cycles	NR	NR	IV and PO Steroids	N	Y
**Francis et al. (2020)**	Ipilimumab and nivolumab	54	F	NR	NSCLC	Bilateral Optic Neuritis; Choroiditis; Intraretinal edema bilateral	After 4 cycles	NR	NR	PO steroids	Y	Y
**Francis et al. (2020)**	Pembrolizumab	69	F	NR	NSCLC	Unilateral Optic Neuritis	After 7 cycles	NR	NR	IV and PO Steroids	NA	Y
**Francis et al. (2020)**	Ipilimumab and nivolumab; Nivolumab	58	M	NR	SCLC	Bilateral Optic Neuritis	After 4 cycles	NR	NR	IV and PO steroids; Plasma exchange	NA	Y
**Fukasawa et al. (2017)**	Nivolumab	69	F	NR	Metastatic lung adenocarcinoma	Myasthenia Gravis	1w after 3d cycle	NR	Y	Iv steroids, Pacemaker	Partially	NR
**Gill et al. (2021)**	Nivolumab	58	F	NR	Metastatic SCLC	Lambert-Eaton myasthenic syndrome	After 2 cycles	Good response	NR	PO steroids, 3,4-DAP, IVIG, rituximab	Partially	Y
**Gutierrez et al. (2021)**	Pembrolizumab	75	M	Filipino	Metastatic NSCLC	Myasthenia Gravis	After 4 cycles	Partial response	N	PO pyridostigmine	Y	Y
**Hibino et al. (2018)**	Pembrolizumab	83	M	NR	Metasatic squamous cell carcinoma	Myasthenia gravis	After 2 cycles	Complete response	NR	Pyridostigmine; PO steroids	Y	Y
**Kim et al. (2019)**	Nivolumab	76	M	NR	Metastatic NSCLC	Myasthenia gravis, myopathy	After 4 cycles	Good response	Y	IV and PO steroids, pyridostigmine, IVIG	Partially	Y
**Lara et al. (2019)**	Pembrolizumab	63	F	NR	Metastatic NSCLC	Myasthenia gravis	After 2 cycles	NR	NR	Systemic steroids, IVIG, pyridostigmine	Partially	Y

NR = not reported, M = male, F = female, Y = yes, N = no, NSCLC = non small cell lung carcinoma, RCC = renal cell carcinoma, IOP = intraocular pressure, RD = retinal detachment, CME = cystoid macular edema, ON = optic nerve, CNV = choroidal neovascularization, RAPD = relative afferent pupil defect, AMN = acute macular neuroretinopathy, ARMD = Age-related macular degeneration, R/ = treatment, PO = per os, IV = intravenous, IVT = intravitreal, IVIG = intravenous immuneglobulines, PLEX = plasma exchange, VTX = vitrectomy

**Loochtan et al. (2015)**	Ipilimumab and nivolumab	70	M	NR	Metastatic SCLC	Myasthenia Gravis	16d after R/ initiation	NR	Y	PO steroids, plasmapheresis; IV steroids, IVIG	Partially	Y
**Lorenzo et al. (2020)**	Pembrolizumab	67	M	NR	Malignant mesothelioma	Myasthenia Gravis	1m after R/ initiation	NR	NR	IV and PO steroids; Pyridostigmine; IVIG	N	Y
**Makri et al. (2020)**	Pembrolizumab	76	M	Caucasian	NSCLC	Bilateral Optic Neuritis	After 3 cycles	NR	NR	IV and PO Steroids	Partially	Y
**Mancone et al. (2018)**	Nivolumab	76	M	NR	Squamous NSCLC	Oculomotor nerve palsy	After 3 cycles	Stable disease	Y	PO steroids	Y	Y
**Mori et al. (2018)**	anti-PD-L1 (MPDL3280A)	64	M	NR	Metastatic NSCLC	Unilateral optic Neuritis	12m after R/ initiation	Good response	Y	IV and PO Steroids	Y	Y
**Nakatani et al. (2018)**	Nivolumab	73	F	NR	Metastatic squamous cell carcinoma	Lambert-Eaton myasthenic syndrome	20w after R/ initiation	Partial response; Progressive cancer	Y	Pyridostigmine; PO steroids	Partially	Y
**Onda et al. (2019)**	Pembrolizumab	73	M	NR	Metastatic lung adenocarcinoma	Myasthenia Gravis	23d after R/ initiation	NR	Y	Systemic steroids	Y	NR
**Phua et al. (2020)**	Durvalumab	66	M	NR	Metastatic lung adenocarcinoma	Myasthenia Gravis	After 4 cycles	NR	Y	PO steroids, PO pyridostigmine; IVIG; mycophenolate mofetil	Y	Y
**Polat et al. (2016)**	Nivolumab	65	M	NR	Metastatic NSCLC	Myasthenia Gravis	After 3 cycles	NR	NR	PO pyridostigmine	Y	NR
**Samanci et al. (2019)**	Atezolizumab	53	M	NR	Metastatic lung adenocarcinoma	Bilateral Optic Neuritis: papilledema, optic disc swelling	After 1 cycle	Partial response	NR	IV and PO steroids	Y	Y
**Sciacca et al. (2016)**	Nivolumab	81	M	NR	Metastatic lung adenocarcinoma	Myasthenia Gravis	After 3 cycles	Progressive cancer	NR	PO steroids	Y	Y
**Tozuka et al. (2018)**	Pembrolizumab	82	M	NR	Pulmonary pleomorphic carcinoma	Myasthenia Gravis	After 3 cycles	Partial response	Y	PO pyridostigmine	NR	NR
**Xing et al. (2020)**	Sintilimab	66	M	NR	Lung adenocarcinoma	Myasthenia gravis, myositis	After 2 cycles	Progressive cancer	Y	IV steroids, IVIG, Plasma exchange, pyridostigmine	Partially	Y
**Orbita and ocular adnexa**												
**Bitton et al. (2019)**	Pembrolizumab	80	M	NR	NSCLC	Bilateral orbital myositis, Bilateral ptosis, OS ophtalmoplegia	After 2 cycles	Complete response	Y	IV Steroids, IVIG, Methotrexate	Y	Y
**Campredon et al. (2018)**	Nivolumab	61	M	NR	NSCLC	Inflammatory ophtalmopathy with ophtalmoplegia (euthyroid)	After 3 cycles	NR	N	IV Steroids	Partially	Y
**Carrera et al. (2017)**	Tremelimumab and durvalumab	68	M	NR	NSCLC	Inflammatory myositis	1m after R/ initiation	NR	Y	PO steroids	Y	Y
**Kamo et al. (2019)**	Pembrolizumab	72	F	NR	Metastatic lung cancer	Myositis	After 2 cycles	NR	Y	IV and PO Steroids	Y	NR

NR = not reported, M = male, F = female, Y = yes, N = no, NSCLC = non small cell lung carcinoma, RCC = renal cell carcinoma, IOP = intraocular pressure, RD = retinal detachment, CME = cystoid macular edema, ON = optic nerve, CNV = choroidal neovascularization, RAPD = relative afferent pupil defect, AMN = acute macular neuroretinopathy, ARMD = Age-related macular degeneration, R/ = treatment, PO = per os, IV = intravenous, IVT = intravitreal, IVIG = intravenous immuneglobulines, PLEX = plasma exchange, VTX = vitrectomy

**Sabini et al. (2018)**	Durvalumab and Tremelimumab	70	M	NR	Advanced lung adenocarcinoma	Graves' orbitopathy	1m after R/ initiation	NR	NR	PO steroids, L-thyroxine	N	Y
**Valenti-Azcarate et al. (2019)**	Ipilimumab and nivolumab	66	M	NR	Metastatic lung carcinoma	Myositis, myocarditis	After 2 cycles	Progressive cancer	Y	IV steroids	Y	Y

Summary of all case reports related to urological cancer.

References	ICI used	Age	M / F	Ethnicity	Cancer	Ocular toxicity	Time to onset	Antitumor efficacy of ICI	Other irAEs	Treatment	Resolution	ICI discontinued
**Renal cancer**												
**Bitton et al. (2019)**	Nivolumab	56	F	NR	RCC	Bilateral panuveitis with serous retinal detachment	After 2 cycles	Stable disease	N	Topical and IV steroids	Y	Y
**Boisseau et al. (2017)**	Ipilimumab; Nivolumab	27	F	Caucasian	Metastatic RCC	Bilateral Optic Neuritis	After 5 cycles	Good response	Y	IV and PO steroids; Plasma exchange	Y	Y
**De Velasco et al. (2016)**	Nivolumab	60	M	NR	Metastatic RCC	Uveitis	After 28 cycles	Complete response	Y	Intraocular steroids	Y	Y
**Francis et al. (2020)**	Atezolizumab	73	F	NR	RCC	Bilateral optic neuritis; Bilateral anterior uveitis	After 95 cycles	NR	NR	PO steroids	Y	Y
**Garibaldi et al. (2020)**	Pembrolizumab	66	M	NR	Metastatic RCC	Ocular Myositis	6w after 4/ initiation	NR	N	Steroids	Y	Y
**Gonzales et al. (2018)**	Nivolumab	66	M	Caucasian	Metastatic RCC	Acute bilateral anterior and posterior scleritis and anterior uveitis	2m after R/ initiation	NR	NR	Topical and PO steroids	Y	Y
**Ho et al. (2020)**	Ipilimumab and nivolumab	57	M	NR	Metastatic RCC	Myasthenia Gravis	After 1 cycle	NR	NR	IV and PO steroids; pyridostigmine; IVIG	Partially	NR
**Lee et al. (2020)**	Ipilimumab and nivolumab	71	M	NR	Stage IV Clear cell renal cell carcinoma	Bilateral granulomatous anterior uveitis, hypotony, nuclear cataracts	1w after initation	NR	NR	Topical steroids and atropine	Y	N
**Noble et al. (2020)**	Nivolumab	61	M	Caucasian	Metastatic RCC	Anterior uveitis (non-granulomatous) OS; cystoid macular edema OS	52w after R/ initiation	NR	NR	Topical steroids	N	Y
**Noble et al. (2020)**	Ipilimumab and nivolumab	42	F	African-American	Metastatic RCC	Anterior uveitis (non-granulomatous); cranial nerve VI palsy	1w after R/ initiation	NR	NR	Topical and IV steroids	NR	Y
**Noble et al. (2020)**	Nivolumab	63	M	African-American	Metastatic RCC	Anterior uveitis (non-granulomatous)	20w after R/ initiation	NR	NR	Topical steroids	NR	Y

NR = not reported, M = male, F = female, Y = yes, N = no, NSCLC = non small cell lung carcinoma, RCC = renal cell carcinoma, IOP = intraocular pressure, RD = retinal detachment, CME = cystoid macular edema, ON = optic nerve, CNV = choroidal neovascularization, RAPD = relative afferent pupil defect, AMN = acute macular neuroretinopathy, ARMD = Age-related macular degeneration, R/ = treatment, PO = per os, IV = intravenous, IVT = intravitreal, IVIG = intravenous immuneglobulines, PLEX = plasma exchange, VTX = vitrectomy

**Peng et al. (2020)**	Pembrolizumab	24	M	NR	Metastatic renal cell cancer	Posterior uveitis with bilateral retinal detachment	3w after R/ initiation	Progressive cancer	Y	IV, PO and topical steroids	NR	Y
**Sagiv et al. (2019)**	Nivolumab	42	M	NR	Metastatic RCC	Thyroid-like ophtalmopathy	2m after R/ initiation	Good response	Y/N	L-thyroxine	Partia lly	N
**Shahzad et al. (2021)**	Ipilimumab and nivolumab	53	M	NR	Metastatic RCC	Bilateral anterior uveitis with elevated IOP	5w after R/ initiation	Stable disease	Y	Topical steroids, Topical timolol, Topical latanprost	Y	Y
**Shahzad et al. (2021)**	Ipilimumab and nivolumab	53	M	NR	Metastatic RCC	Bilateral anterior uveitis	5w after R/ initiation	Partial response	Y	Topical steroids, Topcial cyclopentolate	Y	Y
**Vogrig et al. (2020)**	Nivolumab	76	M	NR	Kidney cancer	Bilateral III cranial nerve palsy (pupil-sparing) < demyelinating polyradiculoneuropathy	After 5 cycles	Progressive cancer	Y	IVIG	Y	Y
**Wang et al. (2018)**	Nivolumab	64	F	Asian	Metastatic RCC	Bilateral panuveitis with serous RD	After 6 cycles (4 months after R/ initiation)	Partial response	N	Topical, IV and PO steroids; periorbital and intravitreal steroid injections	Y	Y
**Yoshida et al. (2019)**	Nivolumab	61	M	Asian	Metastatic RCC	Bilateral anterior uveitis; unilateral vitreous opacity, RPE folds and small amount of SRF	NR	NR	NR	STN, PO and topical steroids; unilateral VTX for vitreal opacity	Y	Y
**Urinary tract/Bladder cancer**												
**Hellman et al. (2019)**	Pembrolizumab and epacadostat	84	M	NR	Stage III urothelial carcinoma	Myositis and myocarditis	After 2 cycles	NR	N	PO steroids; IV steroids	Partia lly	Y
**Kamo et al. (2019)**	Pembrolizumab	78	M	NR	Metastatic urinary cancer	Myositis	After 2 cycles	Progressive cancer	Y	IV steroids, Plasma exchange	Partia lly	NR
**Sagiv et al. (2019)**	Ipilimumab and nivolumab	73	M	NR	Metastatic bladder urothelial carcinoma	Thyroid-like ophtalmopathy	After 3 cycles	Progressive cancer	Y	IV and PO steroids	Y	NR
**Sun et al. (2008)**	Anti-CTLA-4	72	M	NR	Recurrent localized bladder cancer	Bilateral anterior uveitis and ischemic papillopathy	6w after R/ initiation	NR	Y	Topical, PO and IV steroids; mycophenolate mofetil; infliximab; methotrexate	N (atrop hy)	Y
**Takai et al. (2020)**	Pembrolizumab	77	M	Asian	Metastatic bladder cancer	Myasthenia Gravis	20w after R/ initiation	NR	Y	PO steroids; IVIG, dobutamine, carperitide, furosemide; PM	N	Y
**Vanhonsebrou ck et al. (2020)**	pembrolizumab	85	F	NR	Metastatic urothelial cell carcinoma	Corneal graft rejection	After 4 cycles	NR	NR	Topical steroids	Partia lly	Y
**Prostate cancer**												
**Noble et al. (2020)**	Durvalumab	53	M	African-America n	Metastatic prostate cancer	Anterior uveitis (non-granulomatous)	1w after R/ initiation	NR	NR	Topical steroids	NR	N
**Noble et al. (2020)**	Durvalumab	65	M	Caucasi an	Metastatic prostate cancer	Unilateral Optic Neuropathy	NR	NR	NR	IV Steroids	Y	N

NR = not reported, M = male, F = female, Y = yes, N = no, NSCLC = non small cell lung carcinoma, RCC = renal cell carcinoma, IOP = intraocular pressure, RD = retinal detachment, CME = cystoid macular edema, ON = optic nerve, CNV = choroidal neovascularization, RAPD = relative afferent pupil defect, AMN = acute macular neuroretinopathy, ARMD = Age-related macular degeneration, R/ = treatment, PO = per os, IV = intravenous, IVT = intravitreal, IVIG = intravenous immuneglobulines, PLEX = plasma exchange, VTX = vitrectomy

**Parikh et al. (2020)**	Nivolumab	71	M	Caucasian	Prostate carcinoma	Bilateral anterior uveitis with elevated IOP	3m after R/ initiation	NR	NR	Topical steroids, maximal drop therapy	Y	N
**Warner et al. (2019)**	Avelumab	57	M	NR	Metastatic prostate cancer	Sicca Syndrome: mild chronic sialadenitis	30d after R/ initiation	Stable disease	N	None	N/A	Y

Summary of all case reports related to gastrointestinal cancer.

References	ICI used	Age/gender	M/F	Ethnicity	Cancer	Ocular toxicity	Time to onset)	Antitumor efficacy of ICI	Other irAEs	Treatment	Resolution	ICI discontinued
**Conrady et al. (2018)**	Atezolizumab	36	M	NR	Metastatic colon adenocarcinoma	Paracentral acute middle maculopathy, Multiple retinal venous occlusions, intraretinal hemorrhages	2w after R/ initiation	NR	Y	PO steroids	Y	NR
**Emens et al. (2019)**	Atezolizumab	35	M	NR	Metastatic colon adenocarcinoma	Acute macular neuroretinopathy with retinal venulitis	15d after R/ initiation	Progressive cancer	Y	PO steroids	Y	Y
**Kao et al. (2017)**	Pembrolizumab	NR	M	NR	Metastatic Esophageal adenocarcinoma	Autoimmune retinopathy	After 3 cycles	NR	N	IVIG	Partially	N
**Nasr et al. (2018)**	Pembrolizumab	79	M	NR	Metastatic gastric adenocarcinoma	Myositis and myocarditis	After 2 cycles	NR	Y	IV and PO steroids, pyridostigmine, coenzyme Q10 300; plamaphaeresis; methotrexate; PLEX, IVIG	N	NR
**Noble et al. (2020)**	Pembrolizumab	34	F	Caucasian	Metastatic colon adenocarcinoma	Dry eyes, Blepharitis	9w after R/ inititation	NR	NR	Artificial tears	NR	N

Summary of all case reports related to gynaecological cancer.

References	ICI used	Age	Gender	Ethnicity	Cancer	Ocular toxicity	Time to onset	Antitumor efficacy of ICI	Other irAEs	Treatment	Resolution	ICI discontinued
**Breast cancer**												
**Deitch-Harel et al. (2020)**	Nivolumab	43	F	NR	Metastatic breast cancer	Bilateral anterior uveitis	Months after R/ initiation	NR	NR	Topical steroids	Partially	N
**Emens et al. (2019)**	Atezolizumab	32	F	NR	Metastatic breast cancer	Acute macular neuroretinopathy with retinal venulitis	15d after R/ initiation	NR	NR	None	Partially	Y
**Ramtohul et al. (2020)**	Atezolizumab	62	F	NR	Metastatic breast cancer	Bilateral AMN with retinal venulitis	14d after R/ initiation	Progressive cancer	N	NR	Partially	Y
**Uterine cancer**												

NR = not reported, M = male, F = female, Y = yes, N = no, NSCLC = non small cell lung carcinoma, RCC = renal cell carcinoma, IOP = intraocular pressure, RD = retinal detachment, CME = cystoid macular edema, ON = optic nerve, CNV = choroidal neovascularization, RAPD = relative afferent pupil defect, AMN = acute macular neuroretinopathy, ARMD = Age-related macular degeneration, R/ = treatment, PO = per os, IV = intravenous, IVT = intravitreal, IVIG = intravenous immuneglobulines, PLEX = plasma exchange, VTX = vitrectomy

**Gonzalez et al. (2017)**	Pembrolizumab	71	F	NR	Metastatic uterine carcinosarcoma	Myasthenia Gravis	After 4 cycles	Progressive cancer	Y	Steroids	Y	Y
**Sun et al. (2020)**	Ipilimumab and nivolumab	60	F	African-American	Metastatic endometrial carcinoma	Bilateral anterior and intermediate uveitis	22d after R/ initiation	NR	N/R	Topical steroids	Partially	N
**Ovarian cancer**												
**Shahzad et al. (2021)**	Nivolumab	40	F	NR	Metastatic ovarian cancer	Bilateral anterior uveitis	5w after R/ initiation	Partial response	Y	Topical steroids, Topical cyclopentolate	Y	Y

Summary of all case reports related to other cancers.

References	ICI used	Age/ gender	Gender	Ethnicity	Cancer	Ocular toxicity	Time to onset	Antitumor efficacy of ICI	Other irAEs	Treatment	Resolution	ICI discontinued
**Leukemia**												
**Hsiao et al. (2017)**	Pembrolizumab	20	M	NR	Refractory acute myeloid leukaemia	Bilateral acute corneal toxicity: sloughing of the epithelium, inflammatory cells	2w after R/ initiation	NR	Y	Topical steroids, artificial tears, soft contact lenses	Partially	NR
**Hsiao et al. (2017)**	Pembrolizumab	38	F	NR	Refractory acute myeloid leukaemia	Bilateral acute corneal toxicity: corneal erosions	1w after R/ initiation	NR	Y	Topical steroids, artificial tears, soft contact lens, punctum plugs; tarsorrhaphy	Complete response	NR
**Tsui et al. (2020)**	Ipilimumab	49	M	Caucasian	Chronic myeloid leukemia	Unilateral anterior uveitis; unilateral panuveitis with retinal vasculitis and CME	After 4 cycles (3m); after 8m (5m after discontinuation of ipilimumab)	NR	Y	Topical, PO and IVT steroids	Y: OD 20/50, OS 20/20; ERM with lamellar MH	Y
**Glioblastoma**												
**Kartal et al. (2018)**	Nivolumab	9	M	NR	Glioblastoma multiforme	Bilateral Optic Neuritis	After 2 cycles	NR	NR	IV Steroids	Y	Y
**Hodgkin lymphoma**												
**Sun et al. (2020)**	Nivolumab	37	F	Caucasian	Hodgkin's lymphoma	Bilateral anterior uveitis; subretinal fluid, CME and optic nerve leakage on FA	380d after R/ initiation	NR	N/R	Topical and PO steroids	Partially	N
**Cotliar et al. (2016)**	Pembrolizumab	72	F	NR	Stage IV Hodgkin lymphoma	Iritis attributable to sarcoidosis	6m after R/ initiation	NR	Y	PO steroids	Y	N
**Adenoid cystic carcinoma**												

NR = not reported, M = male, F = female, Y = yes, N = no, NSCLC = non small cell lung carcinoma, RCC = renal cell carcinoma, IOP = intraocular pressure, RD = retinal detachment, CME = cystoid macular edema, ON = optic nerve, CNV = choroidal neovascularization, RAPD = relative afferent pupil defect, AMN = acute macular neuroretinopathy, ARMD = Age-related macular degeneration, R/ = treatment, PO = per os, IV = intravenous, IVT = intravitreal, IVIG = intravenous immuneglobulines, PLEX = plasma exchange, VTX = vitrectomy

**Dumbrava et al. (2018)**	Ipilimumab	60	M	NR	Metastatic adenoid cystic carcinoma	Granulomatous inflammation of lacrimal gland	After 2 R/ cycles	Stable disease	Y	None	Y	Y
**Hypopharyngeal cancer**												
**Kikuchi et al. (2020)**	Nivolumab	63	M	NR	Metastatic hypopharyngeal cancer	VKH-like uveitis: papilledema, serous retinal detachment, bilateral anterior uveitis	After 2 cycles	Progressive cancer	NR	IVT steroids; IV and PO steroids	Y	Y
**Leiomyosarcoma**												
**Kao et al. (2017)**	Nivolumab	NR	F	NR	Metastatic leiomyosarcoma	Bilateral internuclear ophtalmoplegia	After 3 cycles	NR	Y	PO steroids	Partially	N
**Merkel cell carcinoma**												
**Park et al. (2018)**	Pembrolizumab	52	M	NR	Metastatic Merkel cell carcinoma	Graves' orbitopathy	After 3 cycles	Partial response	NR	PO steroids, ocular lubricants, oral atenolol	Y	Y
**Thymic cancer**												
**Warner et al. (2019)**	Avelumab	44	M	NR	Metastatic thymic carcinoma	Sicca Syndrome: severe sialadenitis	95d after R/ initiation	Stable disease	Y	PO steroids	Y	Y
**Warner et al. (2019)**	Avelumab	66	F	NR	Metastatic thymic carcinoma	Sicca Syndrome: severe sialadenitis	95d after R/ initiation	Stable disease	N	PO steroids	Partially	Y
**Warner et al. (2019)**	Avelumab	55	M	NR	Metastatic thymic carcinoma	Sicca Syndrome: severe sialadenitis	53d after R/ initiation	Stable disease	Y	None	N/A	N
**Partoid cell cancer**												
**Bitton et al. (2019)**	Atezolizumab	57	F	NR	Parotid adenocarcinoma	Bilateral conjunctivitis	After 10 cycles	Stable disease	Y	Topical and PO steroids	Partially	Y
**Teyssonneau et al. (2017)**	Pembrolizumab	36	F	NR	Metasttic parotid cell carcinoma	DES (Gougerot-Sjogren-like-syndrome)	After 13 cycles	Stable disease	Y	PO steroids, artificial tear drops; pilocarpine chlorhydrate	Partially	N
**Spinocellular carcinoma**												
**Jeyakumar et al. (2020)**	Cemiplimab	86	M	NR	Periocular SCC	Myasthenia Gravis	3w after R/ initiation	NR	Y	IV steroids, Plasma exchange, IVIG	NR	NR

NR = not reported, M = male, F = female, Y = yes, N = no, NSCLC = non small cell lung carcinoma, RCC = renal cell carcinoma, IOP = intraocular pressure, RD = retinal detachment, CME = cystoid macular edema, ON = optic nerve, CNV = choroidal neovascularization, RAPD = relative afferent pupil defect, AMN = acute macular neuroretinopathy, ARMD = Age-related macular degeneration, R/ = treatment, PO = per os, IV = intravenous, IVT = intravitreal, IVIG = intravenous immuneglobulines, PLEX = plasma exchange, VTX = vitrectomy
